# Two-dimensional materials for high density, safe and robust metal anodes batteries

**DOI:** 10.1186/s40580-023-00384-4

**Published:** 2023-08-10

**Authors:** Hoilun Wong, Yuyin Li, Jun Wang, Tsz Wing Tang, Yuting Cai, Mengyang Xu, Hongliang Li, Tae-Hyung Kim, Zhengtang Luo

**Affiliations:** 1 Department of Chemical and Biological Engineering and William Mong Institute of Nano Science and Technology, Hong Kong University of Science and Technology, Clear Water Bay, Kowloon, Hong Kong; 2 Hefei National Research Center for Physical Sciences at the Microscale, University of Science and Technology of China, Hefei, 230026 Anhui China; 3 School of Integrative Engineering, Chung-Ang University, Seoul, 06974 Republic of Korea

## Abstract

With a high specific capacity and low electrochemical potentials, metal anode batteries that use lithium, sodium and zinc metal anodes, have gained great research interest in recent years, as a potential candidate for high-energy-density storage systems. However, the uncontainable dendrite growth during the repeated charging process, deteriorates the battery performance, reduces the battery life and more importantly, raises safety concerns. With their unique properties, two-dimensional (2D) materials, can be used to modify various components in metal batteries, eventually mitigating the dendrite growth, enhancing the cycling stability and rate capability, thus leading to safe and robust metal anodes. In this paper, we review the recent advances of 2D materials and summarize current research progress of using 2D materials in the applications of (i) anode design, (ii) separator engineering, and (iii) electrolyte modifications by guiding metal ion nucleation, increasing ion conductivity, homogenizing the electric field and ion flux, and enhancing the mechanical strength for safe metal anodes. The 2D material modifications provide the ultimate solution for obtaining dendrite-free metal anodes, realizes the high energy storage application, and indicates the importance of 2D materials development. Finally, in-depth understandings of subsequent metal growth are lacking due to research limitations, while more advanced characterizations are welcome for investigating the metal deposition mechanism. The more facile and simplified preparation of 2D materials possess great prospects in high energy density metal anode batteries, and thus fulfils the development of EVs.

## Introduction

**Fig. 1:**
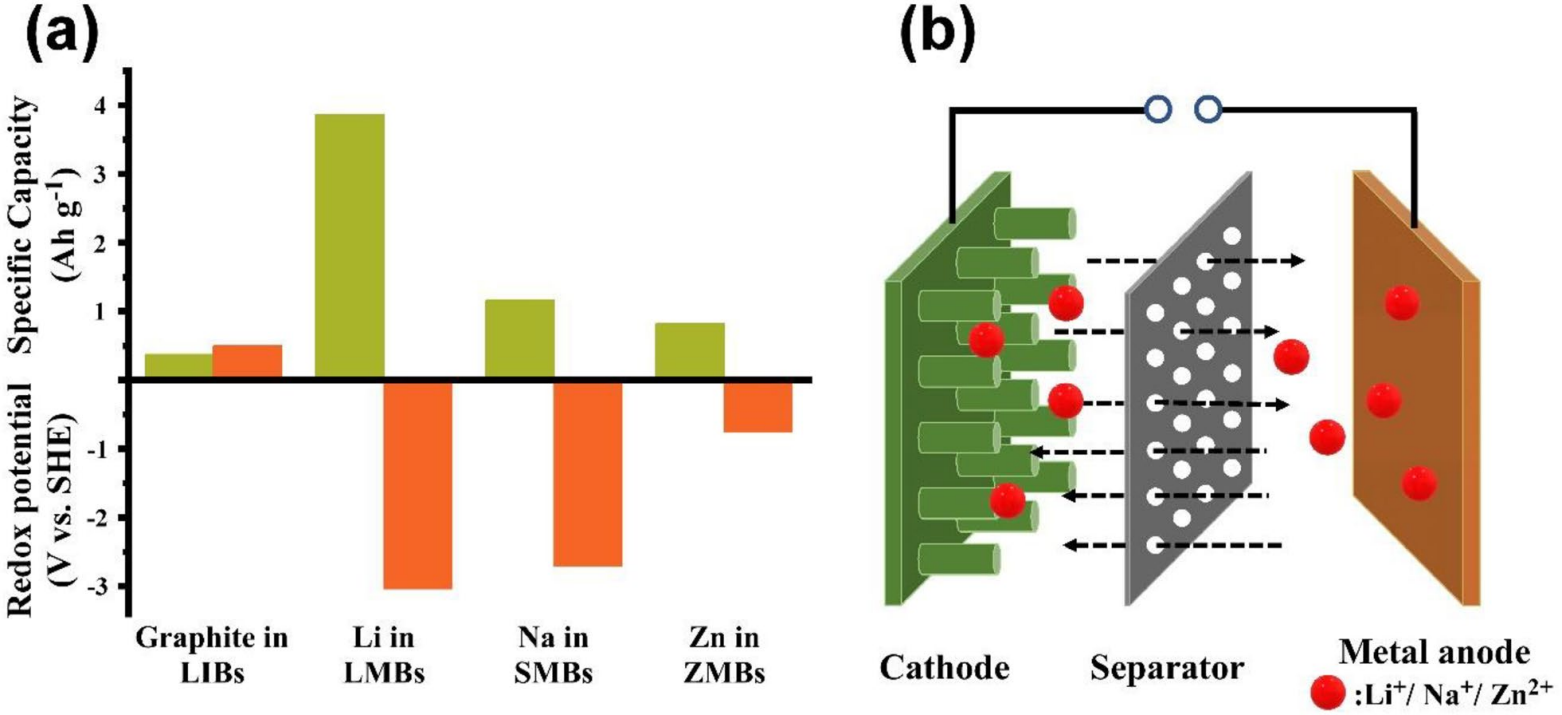
Opportunities and working principles of metal batteries. **a** Comparison between graphite anodes in LIBs, lithium, sodium, and zinc metal anodes in terms of theoretical specific capacity and redox potential. **b** Schematic diagram of the Li/Na/Zn metal batteries

Based on the ion-intercalation mechanism, state-of-the-art lithium-ion batteries (LIBs) have become the most reliable energy storage device, dominating the commercial market for over 30 years, since the first realization by Sony in 1991 [1]. However, the mechanism of inserting Li^+^ ions in graphite anode limits the cell capacity, which makes the LIBs hard to satisfy the energy demand from cutting-edge electronic devices and electric vehicles (EVs) [2–4]. Despite developing more advanced cathode materials, which have increased the energy density of LIBs from 80 Wh kg^−1^ (1990s) to 250 Wh kg^−1^ (most ideal case), the improvement is insufficient and approaching its theoretical limit [5–7]. Therefore, it is imperative to pursue alternative batteries such as metal anode batteries. In Fig. 1a, compared to the conventional graphite anode with a specific capacity of 0.37 Ah g^−1^ and electrochemical potential of 0.5 V vs. standard hydrogen electrode, lithium, sodium and zinc metal anodes (MAs) are regarded as the “Holy Grail” electrodes that receive tremendous research attention, due to their attractive theoretical capacity (3.86, 1.17 and 0.82 Ah g^−1^ for Li, Na and Zn, respectively) and the low electrochemical potential (− 3.04 V for Li, − 2.71 V for Na, and − 0.76 V for Zn vs. standard hydrogen electrode). Furthermore, integration of the MAs with sulfur, oxygen or CO_2_ cathodes, forms the metal–sulfur [8, 9], metal–oxygen [10, 11] and metal–carbon dioxide batteries [12, 13], which are considered as the next-generation energy storage technologies.

**Fig. 2:**
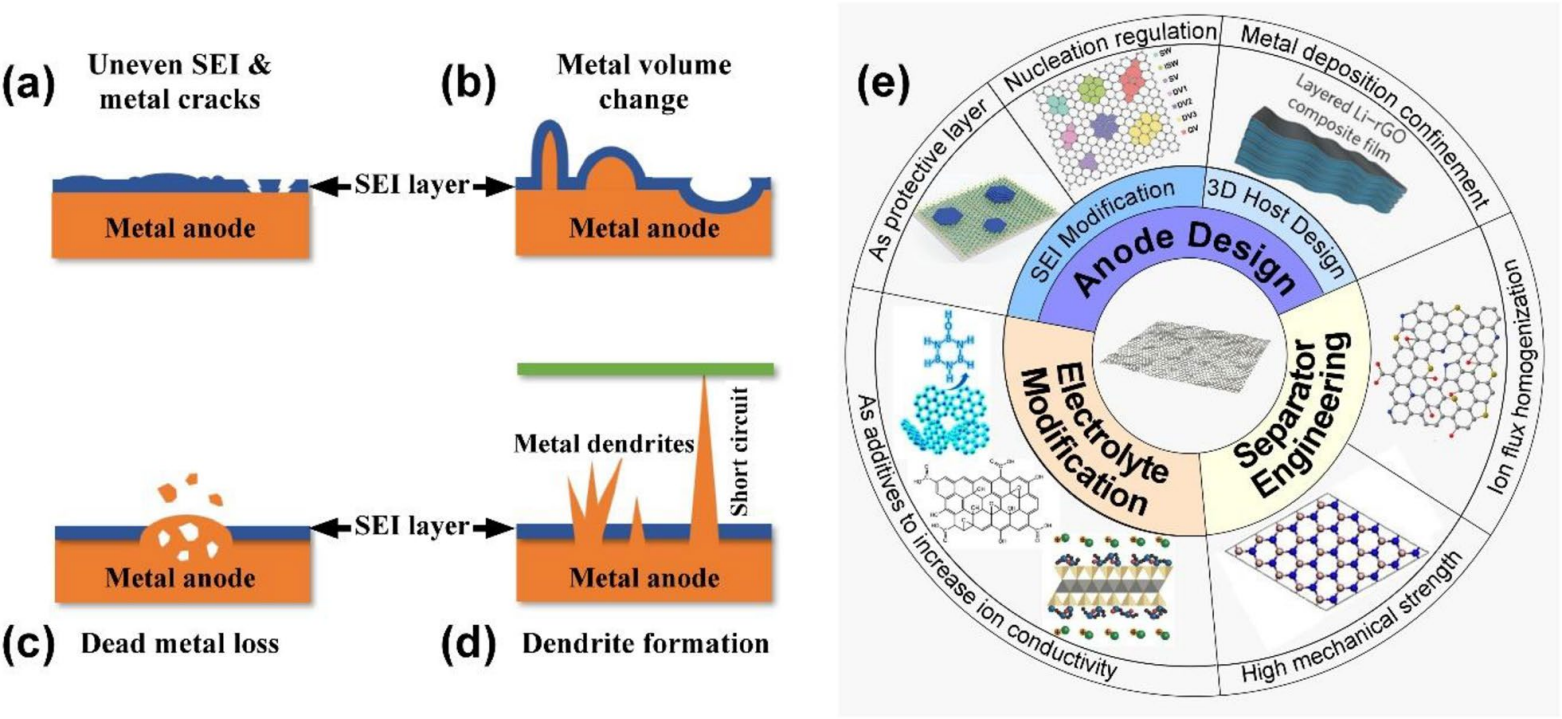
Challenges of anode in metal batteries and the summary of strategies to inhibit dendrite growth. Schematic illustration of metal anode challenges having **a** uneven SEI and cracks, **b** metal volume change, **c** dead metal detached from anode surface and **d** dendrite formation touching the cathode side causing short circuit. **e** 2D materials modification from three aspects of anode design, separator engineering and electrolyte modification

However, the deployment of metal anode batteries is hindered by the safety concerns, arising from forming severe dendrites during the repeating metal ion plating/stripping processes. Figure 1b illustrates the working principles of lithium metal batteries: during the discharge and charge process, metal ions are shuttled between electrodes. More specifically, during discharge, metal ions are extracted from the metal anode and reduced with the cathode materials. The reverse process occurs when the cell is being charged. Figure 2 summarizes the main challenges of dendrite formation in metal anodes. Figure 2a shows that the dendrite growth starts from the uneven metal nucleation, which is highly depending on the inhomogeneity of the substrate surfaces, including anode, current collector, and solid-electrolyte interface (SEI). Rough surfaces with protrusion, cracks and tips can accumulate charges easily and create a nonuniform metal ion flux and charge distribution, allowing metal ion to deposit faster on those area [14]. The newly formed metal nucleation amplifies the surface roughness and thus facilitates the metal deposition into more dendritic structures. The repeating discharge/charge process creates huge volume change at the anode surface and damage the integrity of the SEI and electrode structure (Fig. 2b). The uncontrollable dendrite growth continuously consumes organic electrolytes, leading to poor ionic conductivity, deteriorating the cell performances. In addition, stripping the metal from the dendrite root area may cause extensive loss of active materials, resulting in great capacity loss (Fig. 2c). More importantly, the separator penetration by metal dendrite would cause internal short-circuit, inducing the thermal runaway and explosion, as shown in Fig. 2d. To overcome the obstacles, strategies, including the introduction of electrolyte additives (HF, LiF, H_2_O, and Cs^2+^) [15–17], the design of solid-state electrolyte (SSE) [18, 19], separator engineering [20, 21] and anode surface modification [22], have been devoted to obtaining a uniform metal deposition.

Since the discovery of exfoliated graphene in 2004 [23], 2-dimensional (2D) materials have been extensively studied and explored during the last decade. They are classified as atomically thin nanomaterials in sheet-like structures, stackable via van der Waals forces. 2D materials are ideal building blocks for functional materials. In addition, the in-plane covalent bonds of 2D materials provide strong mechanical strength and structural stability. Together with their unique physical, chemical and mechanical properties, including large surface area, abundant active sites and wide range of bandgaps from insulator to superior electronic conductor, 2D materials, such as, graphene, hexagonal boron nitride (h-BN), transition metal dichalcogenides (TMDs), transition metal carbides/nitrides (MXenes), transition metal oxides (TMOs) and elemental analogues of graphene (silicene, germanene phsphorene, borophene and stanine are promising materials to modulate the properties of metal anodes [24–28]. For example, the basal plane of 2D large area and mono-orientated MoS_2_ demonstrates dendrite-free Zn epitaxial electrodeposition and realizes the practical applications of zinc-ion batteries (ZIBs) [29].

In this review, we summarize the latest development of 2D materials for inhibiting dendrite growth. Firstly, a brief overview of 2D material’s properties and synthesis method will be provided. Then, the major strategies for stabilizing metal anodes using 2D materials will be discussed with examples from both experimental and theoretical points of view in the three aspects of (i) anode design, (ii) separator engineering and (iii) electrolyte modifications (Fig. 2e). Finally, based on the existing challenges, future research direction and potential advancement in the field will be discussed in detail.

## Overview of 2D materials

The unique physical and chemical properties of 2D materials, have made them increasingly popular for electrochemical applications, particularly in battery technology [30]. Several 2D materials, including graphene [31–33], hexagonal boron nitride (h-BN) [34–36], transition metal dichalcogenides (TMDs) [37–39], and transition metal carbides and nitrides (MXenes) [40–42], have demonstrated significant potential as metal anodes in batteries, highlighting their prospects in various electronic applications [35].

### Graphene

Graphene is a highly conducting material that consists of carbon atoms arranged in a hexagonal shape with sp2 hybridization. It has garnered significant attention for its exceptional and intriguing properties, particularly for its ultrathin film characteristics [43, 44]. Over the past few years, there has been a growing research interest in using graphene as electrode materials for energy devices [45–47]. Graphene is an electrode material with high energy and power capabilities, owing to its exceptional electrical conductivity, impressive mechanical flexibility, remarkable thermal conductivity, and large surface area [48–50]. Using graphene as an anode material has the potential to enhance battery performance by improving electron transport rate and enabling reversible specific capacity. Additionally, it is a suitable material for integration into flexible battery development due to its compatibility with such systems. Particularly, heteroatoms doped graphene has illustrated its improvements in electrochemical performance in lithium-ion batteries (LIBs). A strategy for boosting the relative specific capacity (900 mAh g^−1^) and rate performance (250 mAh g^−1^) of LIBs was developed by utilizing the disparity electroneutrality of doped N and B heteroatoms [48]. The disturbance of electrical neutrality in graphene effectively benefits the formation of charge points, thus enhancing the oxygen adsorption rate and discharge performance in LIBs. Coincidentally, another practical approach of phosphorus-doped graphene in LIBs has also been investigated and demonstrated a significant enhancement in specific discharge capacity of about 460 mAh g^−1^ at 0.1 A g^−1^ [32].

### Hexagonal boron nitride (h-BN)

Similar to graphene, h-BN is also in the form of a layered sp^2^ phase with relatively weak bonding in the out-of-plane direction and a robust bonding in the in-plane direction, featuring a honeycomb lattice structure. Interestingly, the remarkable chemical stability of 2D h-BN layers, attributable to their strong intralayer bonds and ultrathin thickness, provides excellent interfacial protection for metal anode, leading to a smooth deposition process that eliminates the dendrite formation. An effective strategy over the h-BN-protected electrodes for metal anodes, showed a significant improvement in cycling stability, current density (2.0 mA cm^−2^), and areal capacity (5 mAh cm^−2^) [34]. In addition, it exhibits high thermal stability and chemical inertness, enhancing the durability of the device. With a high theoretical capacity, it can store a large amount of energy per unit weight, making it highly efficient. It has high electronic conductivity, facilitating efficient electron transfer and reducing resistance, enhancing overall performance [31, 42]. Additionally, hBN has a high specific surface area, enabling a large contact area between the electrode and electrolyte, facilitating ion transfer, and further enhancing the device’s performance [49–54]. Many studies have shown that hBN can act as an effective protective layer at the anode/electrolyte interface in energy storage devices, improving the overall lifetime of batteries [51, 52]. This is due to hBN’s high chemical stability, which helps to prevent unwanted reactions between the electrode and electrolyte.

### Transition metal dichalcogenides (TMDs)

Transition metal dichalcogenides, compositions of MX_2_ (M = Mo, W, Cu, Sn, etc. and X = S, Se, Te, etc.), is another attractive class of layered 2D materials that show huge potentials in the electrochemical field. Introducing defects and doping creates more active sites for metal ion adsorption and storage. TMDs are highly promising anode materials, owing to their unique properties [53, 54]. They exhibit high electronic conductivity, which reduces resistance, leading to fast and efficient electron transfer and better overall device performance [50]. TMDs have high surface area and porosity, facilitating ion transfer and further enhancing device performance. Additionally, they have excellent mechanical properties, such as high flexibility and toughness, making them suitable for use in flexible devices [47, 55, 56]. A promising approach for improving the performance of TMDs as anode materials in metal batteries through the modification of their electronic properties was developed, as demonstrated by the introduction of high valence state Mo species and the construction of Mo–O bonding, resulting in improved maintenance in discharge capability at about 1225.7 mAh g^−1^ after 500 cycles under high current density (1 A g^−1^) [38]. Moreover, a method for synthesizing vertically aligned arrays of 2D ultrathin MoS_2-x_Se_x_ nanoflakes on graphene-like carbon foam was developed and demonstrated the exhibition of superior long-term cycling stability [25, 53, 57–59].

### Transition metal carbides and nitrides (MXenes)

In recent years, there has been significant interest in MXenes as a type of electrode material. The enhanced energy and power density are attributed to the fast and efficient transfer of electrons, due to the high electronic conductivity of MXenes. Furthermore, MXenes are highly stable and resistant to degradation, which makes them suitable for long-term use in metal batteries [60–63]. MXene is a class of layered inorganic materials that is composed of transition metal carbides/nitrides/carbonitrides, firstly reported at Drexel University in 2011. The 2D Ti_3_C_2_ was exfoliated from 3D bulk MAX of Ti_3_AlC_2_ in hydrofluoric acid to selectively remove the Al [64]. The synthesis of V_4_C_3_ MXene from V_4_AlC_3_ through ball-milling treatment, that posses a large specific surface area with the interlayer spacing well-suited for lithium-ion storage was reported [40]. As an anode material in lithium-ion batteries, V_4_C_3_ exhibits excellent capacity, rate capability, and cycling performance, with a specific capacity of 225 mAh g^−1^ after 300 charge and discharge cycles at a current density of 0.1 A g^−1^ and 125 mAh g^−1^ at 1 A g^−1^. Due to their exceptional metallic conductivity, MXenes as anode material hold great promise for high-rate performance in battery field [65, 66]. Among MXenes, Ti_2_CT_x_ and V_2_CT_x_ have demonstrated outstanding rate capability for battery applications, owing to their ultrahigh metallic conductivity of 2.4 × 105 S m^−1^ [67, 68].

## Synthesis method of 2D materials

The unique characteristics of 2D materials are attributed to their dimensions, thickness, and configuration. Hence, the techniques utilized to produce 2D materials have a crucial impact on their practical utility. Typically, there are two approaches to synthesizing 2D nanomaterials: the top-down method [69, 70] and the bottom-up method [71, 72].

### Top-down method

The majority of 2D nanomaterials are obtained from layered compounds held together by van der Waals forces [73], making top-down approaches such as mechanical exfoliation and liquid phase separation, effective in producing 2D materials with clean surfaces and high quality. Researchers have employed the scotch tape or peel-off method to produce defect-free graphene, but this results in low yields [74]. In contrast, exfoliation of graphite oxide or derivatives is more cost-effective and scalable for large-scale production. A scalable and environmentally friendly top-down approach was reported for the synthesis of graphene with high specific surface area and excellent electrochemical properties [75–77]. The approach involved the exfoliation of graphite in a mixture of an ionic liquid and water. The resulting graphene exhibited a high specific surface area, high electrical conductivity, and excellent electrochemical performance as an anode material for Li-ion batteries [78]. Exfoliation is also a widely used technique to produce TMD nanosheets from bulky one. Exfoliation weakens the van der Waals interactions between layers, allowing the easy formation of TMD nanosheets.

Liquid exfoliation involves dispersing the bulk materials in solvents to produce 2D materials. This method is scalable and can produce large quantities of 2D materials. Various solvents such as water, *N*-methyl-2-pyrrolidone (NMP), and dimethylformamide (DMF) have been used for liquid exfoliation. Liquid exfoliation can also introduce functional groups onto the surface of 2D materials by adding chemicals during the exfoliation process. For example, Gao et al*.* reported the exfoliation of MoS_2_ using DMF and ammonium persulfate as a chemical additive, resulting in the preparation of MoS_2_ with oxygen-containing functional groups on the surface [79]. The researchers dispersed graphite powder in specific organic solvents, including DMF and NMP, and subjected it to sonication and centrifugation, which resulted in the production of graphene dispersion [80]. This method is highly fascinating and provides a new opportunity for the cost-effective and large-scale manufacturing of graphene [53]. As a concept, numerous [74, 81] researchers have contributed to the attainment of high-concentration graphene by extending the sonication duration, augmenting the initial graphite concentration, incorporating surfactants and polymers, utilizing solvent exchange techniques, mixing solvents, and so on [82–84]. Two reviews have been published that provide a comprehensive overview of the latest advancements in the production of graphene assisted by sonication [85, 86].

### Bottom-up method

The bottom-up method, particularly chemical vapor deposition (CVD), is a versatile technique for synthesizing 2D nanomaterials through chemical reactions. By regulating parameters of temperature, pressure, carrier gas, time, type of substrate and precursors, the structure of 2D nanomaterials can be precisely controlled. Metal foils are commonly used as the substrates for preparing layered TMD nanomaterials for energy storage applications because the foils can be directly used as electrodes without further transfer processes. For example, WSe_2_ nanowires was synthesized on a W foil as a cathode for magnesium-ion batteries using CVD [87]. In addition to growing pure layered TMD nanomaterials on metal substrates, CVD can be used to grow hybrid electrode materials by growing layered TMD nanomaterials on other electrochemically active materials, such as carbon materials and metal oxides. Choudhary et al. used CVD to grow WO_3_@WS_2_ core–shell nanowires on oxidized W foil by first growing WO_3_ nanowires through oxidation, and then reacting them with S vapor. The resulting WS_2_ shell was around 7–8 nm thick [88]. However, this approach is costly and requires more precise conditions, although it allows the large-scale material synthesis. There are also some reviews on the CVD growth of 2D materials [89–91].

Wet chemical hydrothermal/solvothermal methods are also commonly used for synthesizing 2D materials. These methods involve the reaction of precursor materials in a solvent at high temperature and pressure to produce 2D materials. For example, Zhou et al*.* reported the hydrothermal synthesis of WS_2_ nanosheets using tungsten hexachloride and thiourea as the precursors [92]. Solvothermal methods have also been used for synthesizing 2D materials. Additionally, Song et al. synthesized rGO/WS_2_ composites by hydrothermal method [93]. Compared with self-assembled WS_2_ nanowire microporous spheres (WS_2_ nano-MS), the capacity of graphene/WS_2_ nanohoneycomb (nano-HC) reached about 800 mAh g^−1^ after the charging and discharging cycle at 1 A g^−1^. The rate capability of WS_2_/rGO nano-HC is also better than that of WS_2_ nano-MS. The main function of graphene was to transform the morphology of the microspheres into the planar structure of nanohoneycomb. Liquid exfoliation and wet chemical hydrothermal/solvothermal methods are commonly used techniques for producing 2D materials for use in Li/Na/Zn-based batteries. The synthesis of 1T-MoSe_2_ nanosheets, which were 5–10 nm thick, was achieved by utilizing selenium dioxide (SeO_2_), sodium molybdate (Na_2_MoO_4_), and ethylenediamine (NH_2_C_2_H_4_NH_2_) as precursors through a hydrothermal method at a temperature of 200 °C for a duration of 12 h. These nanosheets exhibited excellent Li ion storage capacity [94]. Behera et al. synthesized 1T-VSe_2_ nanosheets for the application of high-performance supercapacitor electrode, through a hydrothermal method at 200 °C for 24 h using sodium metavanadate (NaVO_3_) and SeO_2_ as starting precursors [95]. The hydrothermal method was primarily utilized to prepare various non-layered TMDs, including NiSe_2_ nanosheets [96], CoS nanowires [97], CoS nanoparticles [98], and CoSe_2_ nanosheets [99, 100], which are challenging to synthesize through the top-down method.

## 2D materials modification for Li/Na/Zn metal anode

### Anode design principle with 2D materials

#### The use of 2D materials as SEI modification layer

In recent years, researchers have been exploring the potential of 2D materials to enhance the performance, safety, and lifespan of metal batteries. One of the most promising applications is to use 2D materials as a modification layer for the solid electrolyte interphase (SEI), a passivation layer that forms on the electrode surface. The optimal SEI can help to stabilize the electrode–electrolyte interface and prevent further degradation from reactions between the electrode and electrolyte. However, if the natural SEI is too thick or fragile, it can lead to electrode degradation and limit battery efficiency. To address this issue, researchers have proposed various SEI modification strategies aimed at forming a strong and robust protective layer above the metal anodes. In this part, we will discuss three categories of 2D materials that have been investigated for SEI adjustment.

#### Graphene and graphene-based materials as functional anode layer

**Fig. 3:**
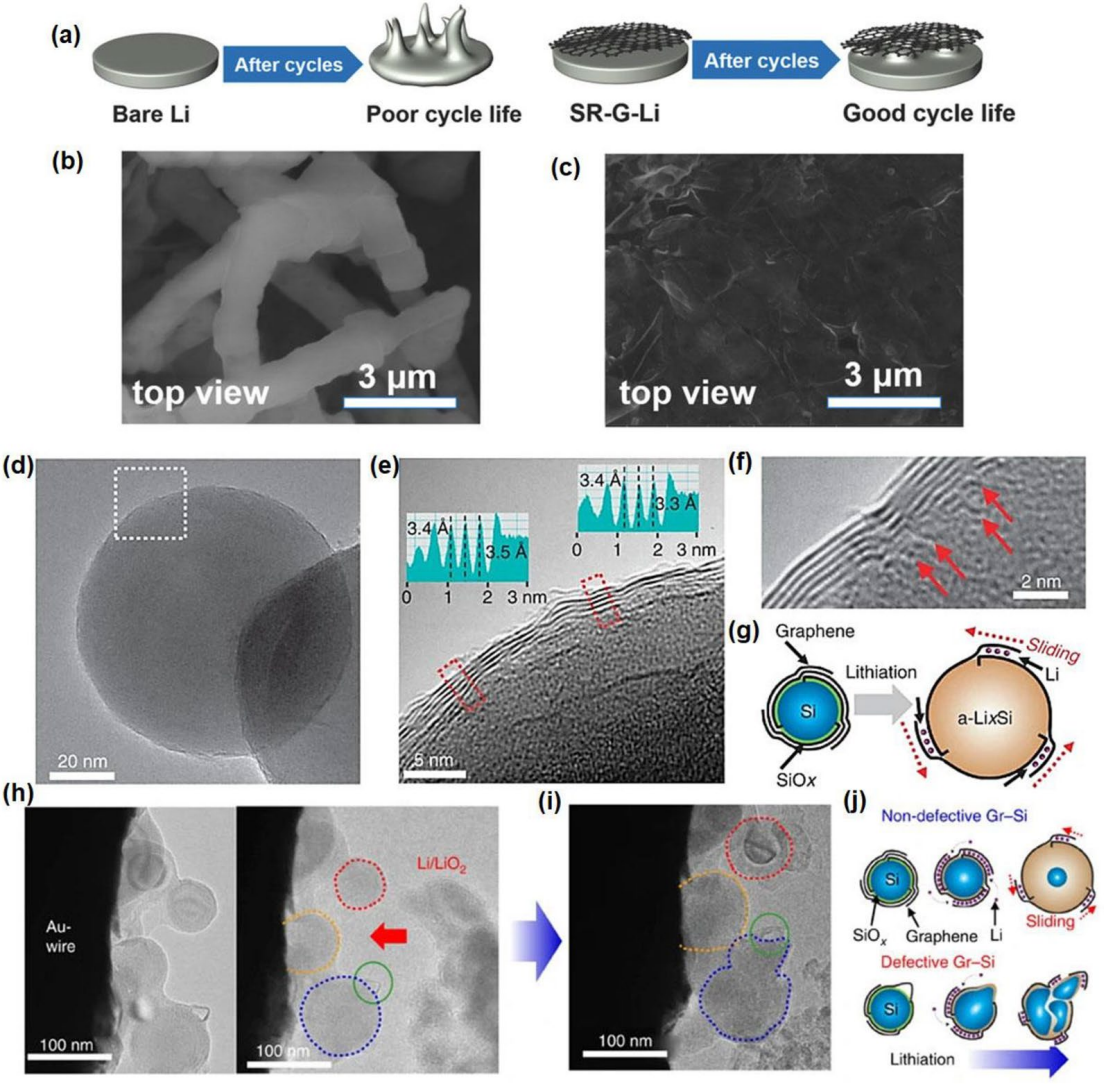
The dendrite prohibition mechanism of several graphene-based materials as SEI protective layer. **a** The Li plating behavior on the pure Li and SR-G-Li anodes. Top-view of the **b** bare Li anode and **c** SR-G-Li anodes. Reprinted with permission from [105]. Copyright 2018, Wiley. **d** TEM image of Gr–Si NP. **e** A higher-magnification TEM image for the same Gr–Si NP, dashed area in **e**. **f** A visualization of the origins (red arrows) from which individual graphene layers grow. **g** The sliding process of the graphene coating layers that can buffer the volume expansion of Si. **h** Gr–Si NPs attached to the surface of Au wire and Li/LiO_2_ electrode. **i** After lithiation. **j** A lithiation process of Gr–Si NPs for both non-defective and defective graphene encapsulation. Reprinted with permission from [112]. Copyright 2015, Springer Nature

With the remarkable electrical conductivity, exceptional flexibility and chemical stability, graphene and graphene-based materials have been conformed to be an exciting artificial layer for the metal anode surface coatings [16, 103, 104, 111]. At the early stage, multiwalled carbon nanotubes are used to serve as the lithium deposition matrix on anode, where the capacity of the graphene electrode outperformed owing to the formation of a more robust SEI [101]. With more functional groups, graphene oxide and the modified graphene were used widely for the batteries, such as the N-doped graphene [102], sulfur-rich graphene [103], p-type dopants [48] or carbonyl, carboxyl, and amine groups-modified graphene [104], which effectuate more active sites and improve the ions mobility. A facial spray method was used to fabricate a spontaneously reduced graphene oxide coating layer on the Li anode, denoted as, SR-G-Li, which significantly improves the electron transfer between the electrode and electrolyte, the stable protective layer can prevent the degradation of the SEI and prolong the lifespan of the battery up to 1000 cycles at the practical cycling condition of 5 mA cm^−2^ [105]. Figure 3 exhibits the examples of graphene-based materials used as the SEI protective layer for efficient dendrite prohibition. As illustrated in Fig. 3a, the Li plating behaviour of the bare Li and SR-G-Li anodes were shown, where the smoother surface achieved above the SR-G-Li after cycling and Fig. 3b, c exhibit the different cross-sectional SEM images of deposited surface with these two different anodes. Moreover, Manthiram’s group [106] found that the conducting polymer PANI decorated graphene exhibited excellent electrons exchanging and transportation. Commonly, the CVD methods such as plasma-enhanced CVD, and chemical vapor reduction for the graphene synthesis were employed in various application area [107]. As for the battery application, we emphasized the hard control of the growth conditions for high-quality graphene, such as pressure, temperature and gas [108]. But some researchers revealed that the existence of defects or other active sites can facilitate Li diffusion and storage [109, 110]. Some DFT calculations suggest that defects and vacancies enable a lower diffusion barrier along the basal plane. Honma et al*.* [110] discovered that the defects of sulfonate groups on graphene sheets can lower the Li deposition barrier for Li accommodation.

Many groups reported the function of silicon-decorated graphene, which can successfully eliminate the volume expansion of the lithiation and delithiation. For example, the layer of Si–graphene was observed to stabilize the anode interface and enhance the Li exchange efficiency with the help of graphene, which is essential for the capacity and performance improvement of battery [111]. From the same perspective, a novel synthesis method for growing graphene directly over silicon nanoparticles (Fig. 3d–f) was presented by Chang’s group [112]. The graphene layers attached to the silicon surface can accommodate the expansion of silicon by sliding between adjacent graphene layers (Fig. 3g) and facilitate the ions transportation simultaneously. As demonstrated in Fig. 3h, Gr–Si NPs get closed to the Au wire surface, once interacting, the particles began to swell (Fig. 3i). Figure 3j explains two expanding structures, which contributed 972 and 700 Wh l^−1^ volumetric energy densities at first and 200th cycle, much higher than the commercial LIBs, proving that the surface chemistry has been proposed as a contributing factor to the capacity of chemically driven graphene sheets [113].

In addition to the benefit of ion mobility and active sites enhancement, the modified graphene SEI also plays a vital role in mechanics improvement. The LiF-graphene layer was proposed by Cheng’s group [114]. They put forward that LiF-modified graphene served as an electrochemically stabilizer with high shear modulus for the high-power LIBs which functionally inhibits the side reaction of LiFP_6_ decomposition, showing excellent rate reversibility and significantly improved cell stability and CE [114]. Similarly, they reported the lithiation behaviour of layered graphene nanoribbons via in situ transmission electron microscopy (TEM). A Li_2_O layer was discovered to form on the graphene surface during the lithiation, but it cannot be removed on the charging process, which indicated the Li_2_O/graphene composite layer existed on the anode surface serving as the stable SEI layer with the great flexibility [115]. In addition, NaF is recognized for its chemical stability in electrolyte solvents, which helps reduce side reactions for the sodium metal batteries. NaF/C-F protective coating was proposed by our group, which possesses exceptional mechanical strength that hinders dendrite growth, resulting in improved battery cycling performance and safety [116].

The first time mentioned the extremely low lattice mismatch between graphene and Zn, as demonstrated in Archer’s related works [117], the deposited Zn tends to from layer-by-layer hexagonal Zn crystal during the epitaxial deposition above the graphene interface owing to the well lattice match between Zn and graphene. The ZIBs with graphene modified electrode achieves relatively high reversibility and stability. Until now, lots of the research proposed the epitaxial mechanism with lattice mismatch between Zn and artificial layer, which will be fully discussed in the TMDs parts below.

Above all, we need to realize some limitations of the CVD methods for the large-scale application, such as high cost since CVD methods require expensive equipment and controlled environments; hard quality control since the quality of the graphene-based materials synthesized by CVD methods can be influenced by a range of factors, including the growth conditions, the choice of substrate, and the quality of the precursor materials. This can make it challenging to achieve consistent quality and reproducibility in large-scale batteries production.

#### Typical MXenes as functional anode layer

With abundant functional chemistry surfaces, high conductivity, and the ability to constructure macro-structure, MXenes also serves as a promising candidate for the anode surface modification [118, 120–122]. Several studies have shown that the use of MXenes as SEI modification layers can enhance the performance of various types of metal batteries, including lithium-ion batteries and sodium-ion batteries. Briefly speaking, the aim for designing the ideal MXene coatings for metal anode modification, can be divided into three points [118–120]. Firstly, MXene layer can improve the lithiophilicity or sodiuphilicity, which guarantees the abundance of adsorption sites for metal deposition. Secondly, the promising MXene candidates enhance the ions conductivity and homogenous distribution, which help to achieve the even deposition morphology. Finally, the expandable layers relieve the volume change during the repeated cycling and protect the metal anode from continuous side reaction, resulting in the high capacity of the cells and stable cycling. Overall, the new family of 2D materials, MXene, also gains great attention and has been widely used in the energy storage system.

MXene with active sites plays a vital role in lowering the nucleation barriers and improving the metal wettability for the metal deposition. For instance, Zn single atom decorated MXene (Zn–Ti_3_C_2_Cl_x_) layers [118] were produced in a large scale to enhance the nucleation homogeneity and growth of Li seed on the Li anode surface, where the lithium tends to grow horizontally around the abundant active Zn sites with the lower nucleation barriers. In addition, parallelly aligned Ti_3_C_2_T_x_ (called PA-MXene) layers with the inherent fluorine terminations can serve as a durable artificial SEI for LMBs. From their work, it evidents that the PA-MXene layer facilitates the Li horizontal growth during the deposition due to the favourable binding between F groups and Li, which will guide the uniform nucleation of Li in the electrochemical test, as the cells with the decorated MXene layer delivered a longer life compared with the neat counterpart [121]. Besides, thanks to the excellent conductivity and expanded space between two layers, Niu’s group utilized the self-exfoliated MXene stacks for Li smooth plating/striping to reveal the homogenous charge distribution. Both the full and asymmetric cells exhibited a high and stable discharge capacity under the current density of 10 mA cm^−2^ loading [122]. Na_3_Ti_5_O_12_-MXene hybrid nanoarchitecture which composed of Na_3_Ti_5_O_12_ between Ti_3_C_2_ MXene nanosheet, was synthesized for the sodium metal batteries. The strong chemical interaction between Na_3_Ti_5_O_12_ and Na results in the lower Na reduction barriers and even homeless Na distribution. As a result, the hybrid MXene exhibits high CE with 98.8% for over 200 cycles and stable cycling [119].

**Fig. 4:**
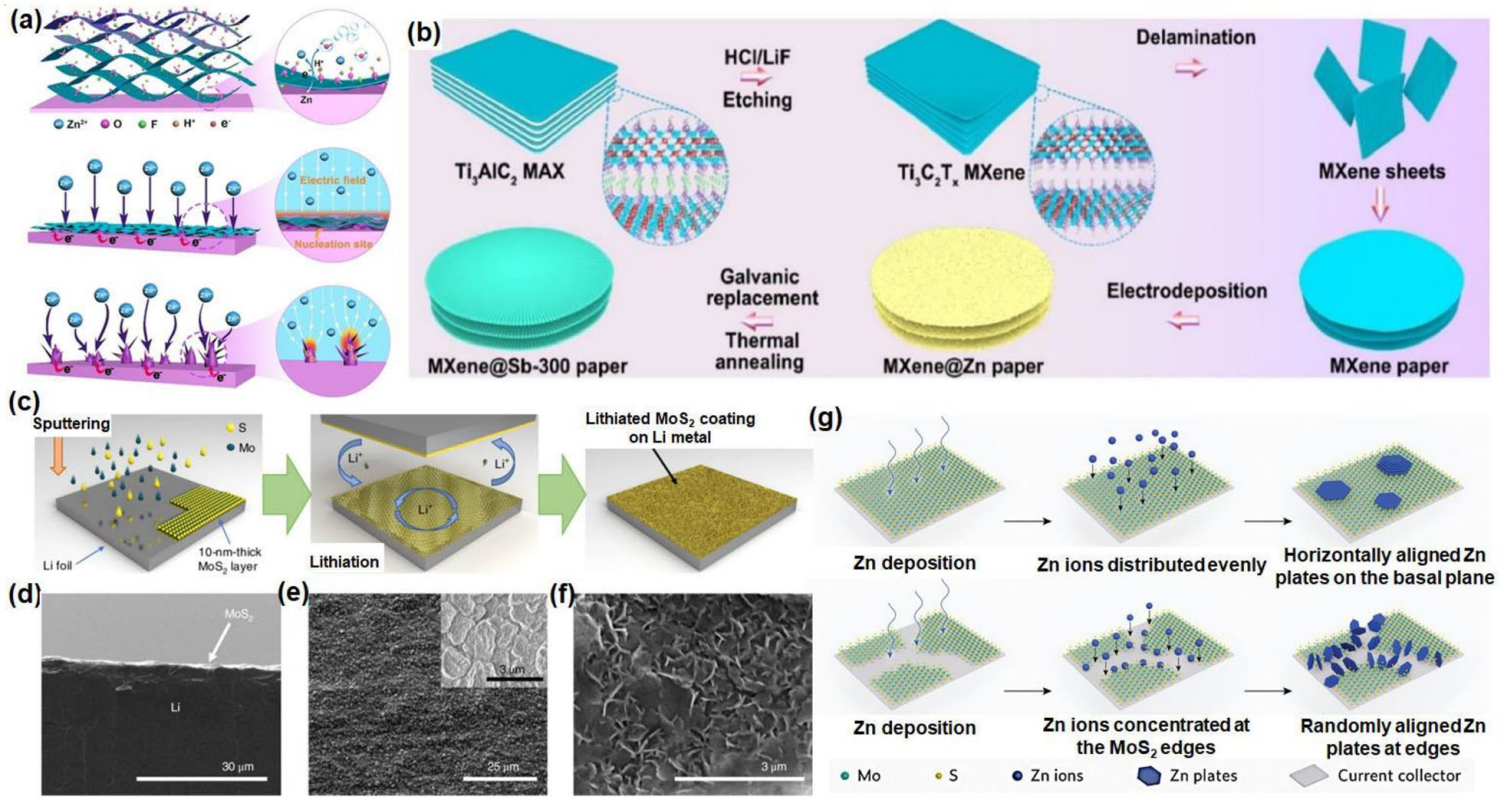
The dendrite prohibition mechanism of several MXene, TMD-based materials as SEI protective layer. **a** Illustration of Zn plating process on MXene-coated Zn with dendrite-free deposition, and pure Zn with pronounced intrusions. Reprinted with permission from [120]. Copyright 2020, Wiley–VCH GmbH; **b** preparing process of MXene@Zn paper serving as anode. Reprinted with permission from [124]. Copyright 2021, Elsevier; **c** the fabrication process for a MoS_2_-coated Li anode via sputtering and subsequent lithiation. Cross-section (**d**) and top view (**e**) SEM images of the as-deposited MoS_2_ on Li metal. **f** Top view SEM image of the lithiated MoS_2_ on Li-metal surface. Reprinted with permission from [126]. Copyright 2018, Nature Nanotechnology. **g** The possible competitive reaction pathways of Zn deposition on basal plane and edges of MoS_2_ substrate. Reprinted with permission from [29]. Copyright 2022, Wiley–VCH

As for the ZIBs without a natural SEI, the MXene-based artificial SEI layer tends to more useful for the Zn batteries for the prolonged cycling. As proposed by Niu’s researchers, the self-assembly ultra-thin MXene on the Zn anode also can lower the Zn deposition barriers and provide a well-distributed electric field for uniform Zn arrangement, which contributes to the dendrite-free Zn anode, the deposition mechanism is illustrated in Fig. 4a [120]. In addition, other kinds of MXene like zincophilic MXene@oxides [123], Zn@MXene@antimony (Fig. 4b) [124], etc. serve the same function for the Zn even deposition.

As summary, MXenes show great potential as electrode coating materials owing to its intrinsic properties of having abundant metal ion adsorption sites, high ion conductivity, and expandable material structure. However, there are various limitations still hindering the practical limitations that MXenes exhibit poor adhesion to the electrode substrate that may result in material delamination and poor cycling stability. Secondly, the production of MXenes is currently limited to laboratory scale synthesis that make it challenging to meet the large quantity fabrication in commercial applications. While limited understanding of MXenes properties limits their practical application, despite significant research progress has been made in the field of MXenes.

#### Typical TMDs as functional anode layer

Apart from the graphene-based materials, TMDs which are composed of chalcogens (S, Se or Te et al.) and transition metals, are also widely used as the functional layer for the metal anode moderation to achieve the uniform metal nucleation. TMDs have been widely considered as attractive cathode materials due to the favourable intercalation properties, high ionic conductivity and mechanical stability [125–127]. And this part will provide an overview of the current state of research on TMDs as protective layers on the anode surface in lithium, sodium, and zinc batteries, which is also desirable for high-energy-density batteries with prolonged cycling lifespan.

Cha’s group reported the ultrathin 2D molybdenum disulfide (MoS_2_) utilized as a functional coating for lithium metal anodes in batteries [126], the sputtering fabrication method is presented in Fig. 4c. It was found that Li atoms intercalated into the layered MoS_2_, the MoS_2_ coating significantly facilitates the flow of Li and forms the stable interface, which delivered a high cycling stability and prevented the formation of dendrites on the metal anodes. The SEM images from cross-section (Fig. 4d, e) and top view (Fig. 4f) showed the dense lithiated MoS_2_ on Li-metal surface [126]. Vertically aligned Tungsten disulfide (WS_2_) thin film was constructed on the Al foil as the Na anode materials. They found that the WS_2_/C film enables high ion and electron transportation and serves as protective layer, preventing the natural SEI from continuous cracking [127].

In addition, the adjustable defects or vacancies from various etching and post-treatment methods make a difference to the intrinsic electrical and catalytic properties of the TMDs [128]. For example, nitrogen doped WS_2_ ultrathin nanosheets were employed in the lithium anode [129]. The synergistic effect of abundant Li attractive sites and layered hierarchical structure enhances the specific capacity up to 801.4 mAh/g and great capacity retention of 95.8% after 150 cycles. Another published example of TMDs as protective layer, is the use of the TiS_2_ due to its simple synthesis and high surface area [130]. The authors explored one general CVD method to prepare thin‐walled tubule of TiS_2_ coated current collector. The microtubular TiS_2_ electrode exhibited lower resistance and high electron transference.

Some TMDs were discovered to modify the nucleation and growth of crystalline Zn metal. The epitaxial growth mechanism in batteries have been intensively proposed for the SEI-free ZIBs [117]. For instance, inspired by the epitaxial growth, the MoS_2_-mediated interface was used for the Zn batteries [29]. The perfect mono-orientated MoS_2_ films without defects was considered as an effective substrate layer for hexagonal Zn formation, suppressing the metal dendrites finally. Figure 4g illustrates the competitive reaction pathways of Zn electroplating above a well basal plane and edge sites of MoS_2_ substrate, where the dendritic Zn appears a lot at the edge sites, whereas the soother morphology appears on the basal plane. Our group induced the 1T-VSe_2_ as the artificial protection layer for the Zn metal anode [131], which lessened the side reaction and regulate the Zn deposition and nucleation. The DFT simulation study have confirmed the lowest adsorption energy between VSe_2_ and Zn, the classic MD studies mimicked the deposition process at the atomic scale, proofing that the excellent Zn mobility above the VSe_2_ which facilitates the regular Zn atom distribution and (002) plane formation due to the thermodynamically energy minimization. The artificial VSe_2_ results in the relatively smooth and dendrite-less anode surface.

Although TMDs show promise as protective layers in batteries, they are still facing limitations that need to be addressed, including optimizing the synthesis and design of TMDs for use, and improving their compatibility with battery components (Table 1).

**Table 1:** The comparison of electrochemical performances for metal batteries using 2D materials as artificial functional layers on anodes

Materials	Types of battery	Working condition	Cycling performance
Graphene-based nanosheets [103]	LIBs	1488 mA g^−1^ (4 C)	500 cycles
reduce graphene oxide [104]	LMBs	5 mA cm^−2^	1000 cycles
Si–graphene [111]	LMBs	0.1 C	1307 mAh g^−1^; 89% retention after 50 cycles
Ti_3_C_2_Cl_x_ [118]	LMBs	1.0 mA cm^−2^	Nucleation overpotential of 11.3 mV; 1200 h
Ti_3_C_2_T_x_ [121]	LMBs	1 mA cm^−2^	1000 cycles
ILC-Li [122]	LMBs	10 mA cm^−2^, 10 mAh cm^−2^	Overpotential of 135 mV, 1050 cycles
MAX (Ti_3_AlC_2_) phase [120]	ZIBs	0.2 mA cm^−2^	47 mV for 400 cycles; 81% capacity retention after 500 cycles
2D MoS_2_ [126]	LSBs	0.5 C	1200 cycles, CE of ~ 98%
WS_2_ [127]	SIBs	50 mA g^−1^	87.5% retention of the initial performance after 50 cycles

#### 3D host design

**Fig. 5:**
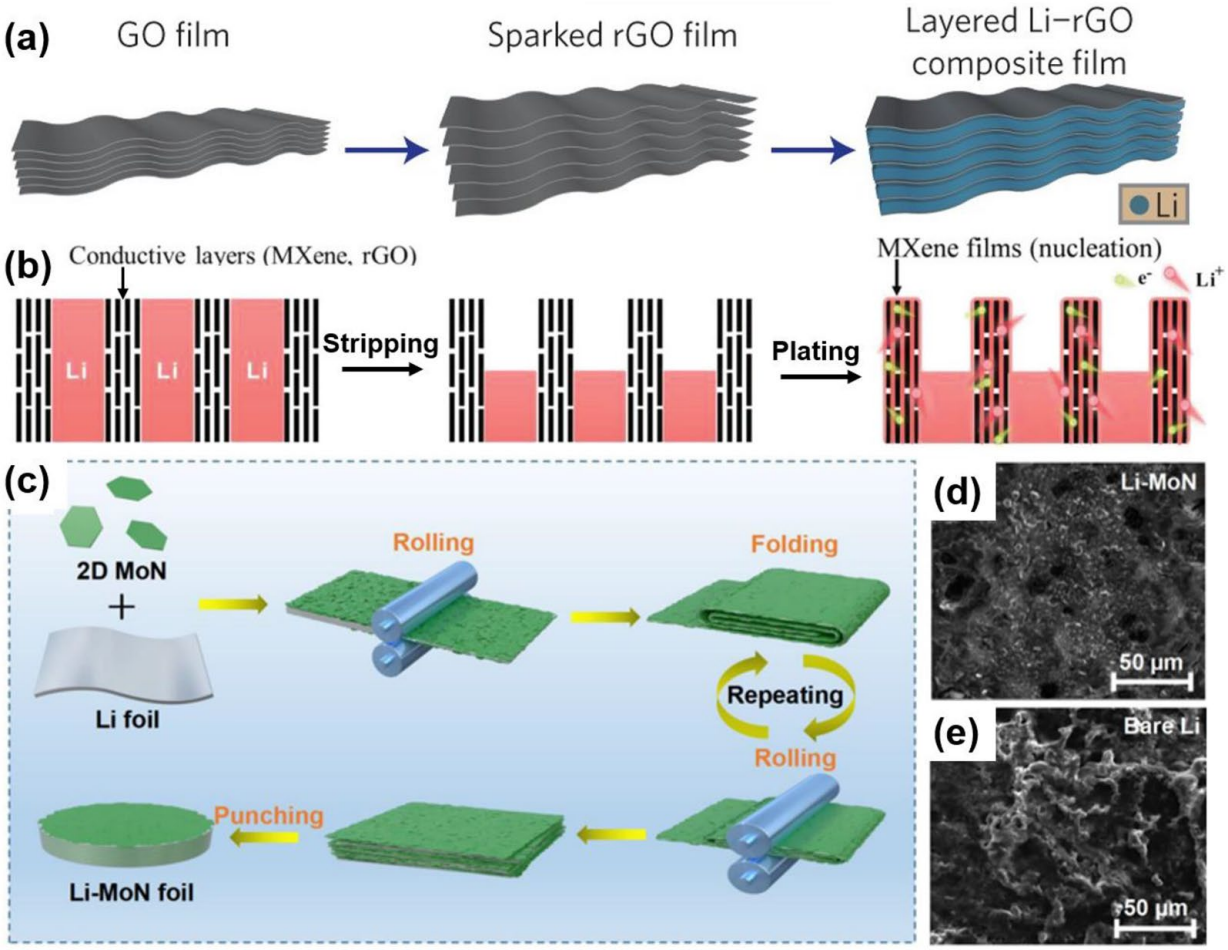
2D materials composited Li metal anodes. **a** Schematic of the material design and the consequent synthetic procedures from a GO film (left) to a sparked rGO film (middle) to a layered Li–rGO composite film (right). Reprinted with permission from [134]. Copyright 2016, Nature Publishing Group. **b** Schematic illustration of the stripping and plating states of perpendicular MXene–Li and rGO–Li arrays. Reprinted with permission from [135]. Copyright 2020, Wiley–VCH. **c** Schematical diagram of the fabrication process of the Li-2D MoN composite anode; SEM images of Li–MoN anode (**d**) and Li anode (**e**) after 30 cycling tests. Reprinted with permission from [136]. Copyright 2023, Elsevier. 2D materials composited Li metal anodes can effectively alleviate volume change

To alleviate the infinite volume change of metal anodes, confining the metallic Li/Na into 3d porous architectures to obtain the composited metal anodes is a promising strategy. 2D graphene-based materials and MXene are the most comprehensive studied materials, owing to its effective surface area and abundant function groups [24, 132, 133]. For example, researchers developed a layered reduced graphene oxide (rGO) structure with uniform nanoscale gaps to confine the lithium deposition through thermal infusion method, as shown in Fig. 5a [134]. The final Li–rGO anode was composed of 7 wt% lithiophilic layered rGO film and could exhibited a high capacity of 3390 mAh g^−1^, along with low overpotential in a carbonate electrolyte. The layered rGO could not only react as a electrochemically and mechanically stable scaffold to regulate the lithium deposition and prevent the volume change during charging/discharging, but also contribute to stabilizing the as-formed SEI. Moreover, Cao et al. proposed to synthesize controllable assembly 3D Ti_3_C_2_–MXene–Li arrays with abundant nanometer-scale and micrometer-scale interspaces [135]. As shown in Fig. 5b, lithium ions travelled fast through plenty of vertical short channels in the composite Ti_3_C_2_-MXene host, thus homogenizing the current density distribution and lithium flux. The abundant internal channels also provided enough space for metallic lithium accommodation and could effectively reducing the volume change of Li during cycles. More recently, Gao et al. synthesized a lithiophilic and conductive framework consisting of 2D molybdenum nitride (MoN) nanosheets, which is 240 μm in thick [136]. Different from the strategies using molten metal infusion approach, the MoN nanosheets powder was firstly synthesized from a chemical reaction and freeze drying. Then, a simple mechanical rolling and folding method was applied, that is folding and rolling the as prepared 2D MoN nanosheets with Li foil for several times to get the composite metal anode. The MoN nanosheets would be embedded and attached strongly with the lithium metal during the repeatedly folding and calendaring process, as shown in Fig. 5c. Meanwhile, the Li was confined in the lamellar 2D MoN films integrated structure with good affinity and rich interfaces. The 2D MoN nanosheets in the composite anode was finally evaluated to be about 50 wt%. The 2D MoN nanosheets with excellent conductivity could not only endow the whole framework with lithiophilic nature, but also inhibit the perpendicular dendrite growth due to the negligible lattice mismatch with lithium crystal. The interwoven scaffold also provided large amount of Li nucleation sites so that the Li grew axially to fill the nanoscale gaps between the 2D MoN nanosheets, thus preventing volume change during charge/discharge (Fig. 5d, e). Moreover, the in situ formed Li_3_N from the reaction between MoN nanosheets and Li atoms boosted ionic conductivity and stabilized the interface. In all, the Li–MoN anode achieved a planer Li plating morphology and excellent cyclic stability for over 2500 h at 1 mA cm^−2^, as well as high Coulombic efficiency within 650 cycles in full cells.

**Fig. 6:**
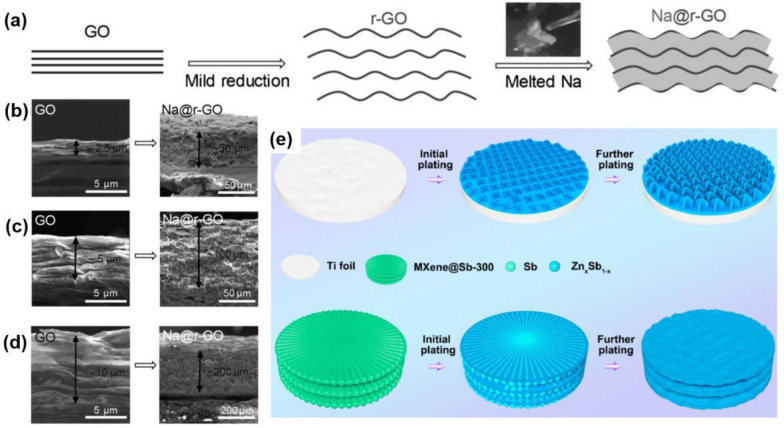
2D materials composited Na/Zn metal anodes. **a** Schematic representation of the preparation of Na@r-GO composites. Densely stacked GO films can expand greatly owning to the gas evolved during the reduction reaction. Upon contact with r-GO, the melted Na can be absorbed into the space between the r-GO sheets. **b**–**d** SEM images of GO films with different thickness and their corresponding Na@r-GO composite films, whose thickness are roughly 20-times of that of GO. Reprinted with permission from [137]. Copyright 2017, Wiley–VCH. **e** Schematic representing Zn deposition behaviour on MXene@Sb-300 and Ti foil. Reprinted with permission from [124]. Copyright 2021, Elsevier. 2D materials composited Na/Zn metal anodes can also homogenize the metal deposition

Similarly, except for Li, in the other metal battery systems, such as Na, Zn, etc., 2D materials composited metal anodes are regarded as an effective way for anode protection. In the study of Wang et al., they proposed a processable and mouldable composite Na metal anode by using reduced graphene oxide (r-GO), as shown in Fig. 6a [137]. The composite anode, where r-GO only took less than 5 wt%, exhibited incredibly improvement in hardness, strength, and corrosion resistance compared with pristine metallic sodium. The layered r-GO provided space for Na accommodation and was contributed to less dendrite growth, while the composite Na metal anode maintained the deformation ability to be proceeded to different shapes and sizes (Fig. 6b–d). What’s more, the cyclic performance of the composite anode was superior in both ether and carbonate electrolytes. In another study, uniform antimony (Sb) nanoarrays were grown on Ti_3_C_2_T_x_ MXene paper to use for dendrite-free zinc-based anodes in aqueous zinc batteries, as shown in Fig. 6e, f [124]. It was proved that ZnSb alloy would form during the cycles originated from the reaction between Zn and Sb, which was able to storage Zn ions during alloy process as well as provide nucleation sites with high Zn affinity to homogenize Zn plating (see Fig. 6g). The freestanding MXene@Sb electrode could effectively suppress Zn dendrite growth and ultimately delivered a long cyclic lifespan up to 1000 h.

3D host structured has been explored as one of the potential solutions to suppress the dendrite formation in metal batteries. Nevertheless, the fabrication complexity increases the cost and limits the scalability of large-scale manufacturing processes, while the structure compatibility and mechanical stability of the 3D host structures remain unsolved. More research efforts are needed in this field.

### Separator engineering

The separator is one of the most important cell components in batteries that mainly functions in separating the electrodes to avoid direct contact and internal short-circuit, while at the same time permitting the ion transport and remaining electronic insulating. Currently, the most widely used separators are polyethylene (PE) and polypropylene (PP) in LIBs, glass fibre in LMBs. However, they can be punctured easily by metal dendrites. Functionalizing the separator using 2D materials enables a higher mechanical strength, better structure flexibility, facile electrolyte permeation and increased chemical and thermal stability [138], and therefore inhibits the dendrite growth by blocking the dendrite growth, regulating the metal ion nucleation and deposition and distributing the metal ion transport. In this part, we summarize the works of using three categories of 2D materials for separator modifications.

#### Graphene and graphene-based materials as functional separator

**Fig. 7:**
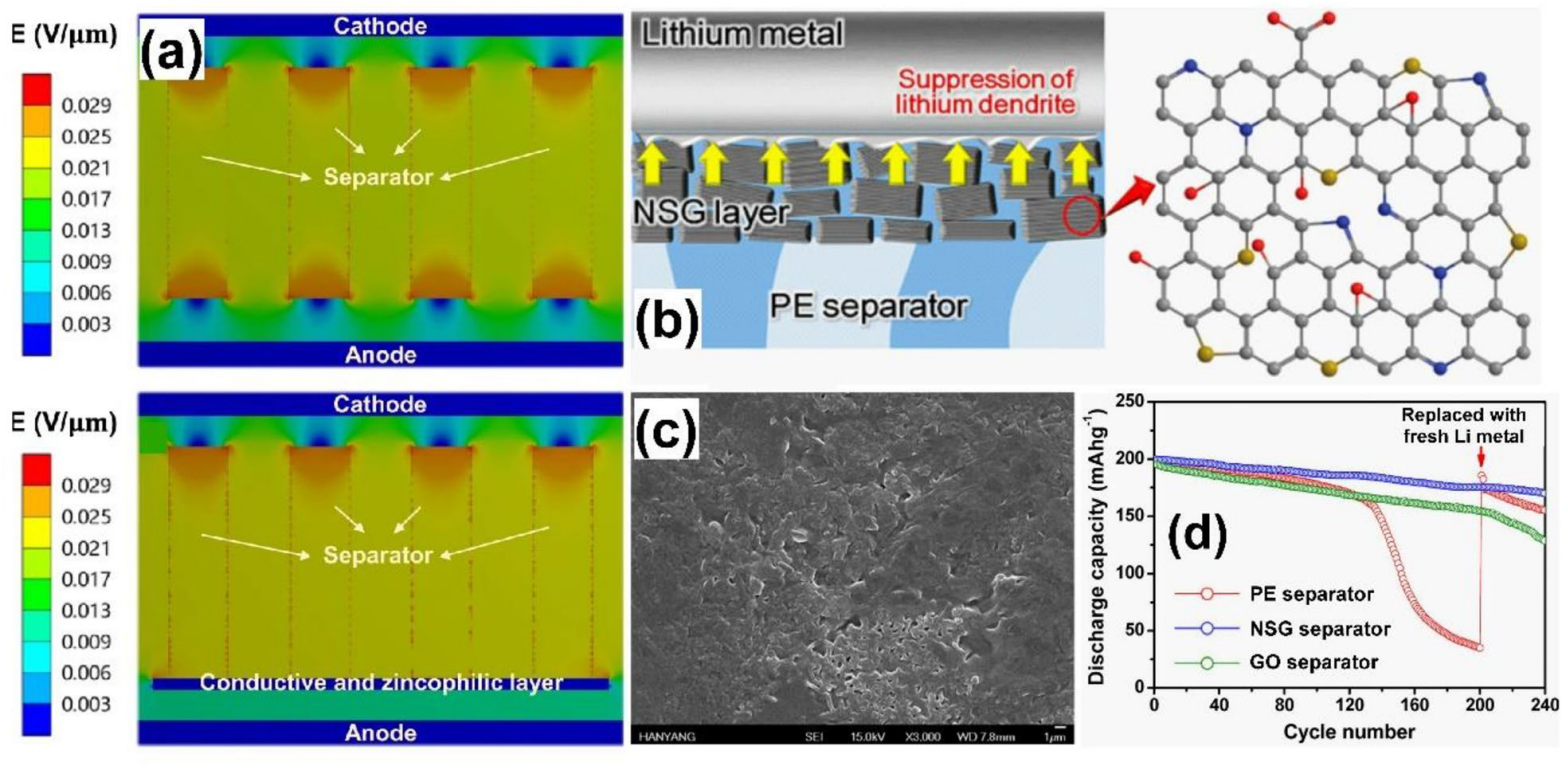
Carbon coating on separator to homogenize electric field and ion flux for dendrite-free metal anodes. **a** Theoretical simulation of electric field distribution in pristine (upper) and modified (lower) separators. Reprinted with permission from [139]. Copyright 2011, Springer Nature. **b** Schematic illustration of separator with NSG coating for suppressing the lithium dendrite growth. **c** SEM image of lithium electrode with NSG separator after 200 cycles, showing smooth lithium deposit. Reprinted with permission from [142]. Copyright 2015, American Chemical Society. **d** Cycling performance of the Li/LiNi_0.8_Co_0.15_Al_0.05_O_2_ cell at 0.5C rate. Reprinted with permission from [143]. Copyright 2020, Wiley–VCH

As illustrated in Fig. 7a, due to the uneven electric field distribution of separator, metal ions tend to accumulate at the protuberances with high surface energy, which are the pores of the membrane, and thus results in inhomogeneous metal deposition forming dendrite [139–141]. An effective separator coating endows a uniform ionic flux on the metal surface, enabling a smooth metal deposition. Kim and coworkers introduce the composite of nitrogen and sulfur codoped graphene (NSG) on polyethylene (PE) by simple vacuum filtration that effectively suppress the Li dendrite growth by maintaining a uniform ionic flux on the anode surface (Fig. 7b, c). In addition, the heteroatom doping on the graphene enhanced the interfacial interaction between NSG and metal surface and the enhanced thermal stability remarkably improved the electrochemical performances [142] (Fig. 7d). Li et al. [143] subsequently developed the vertical graphene (VG) on commercial glass fiber by Plasma enhanced chemical vapor deposition (PECVD) with the oxygen and nitrogen doping via air plasma treatment, render high surface area with even electric field distribution, favourable for building up a uniform Zn ionic flux and therefore, stabilized the Zn anode and achieved 93% cyclic retention over 5000 cycles at high current rate of 5 Ag^−1^ for capacitor and energy density of 182 Wh kg^−1^ in the V_2_O_5_/Zn full cell configuration.

**Fig. 8:**
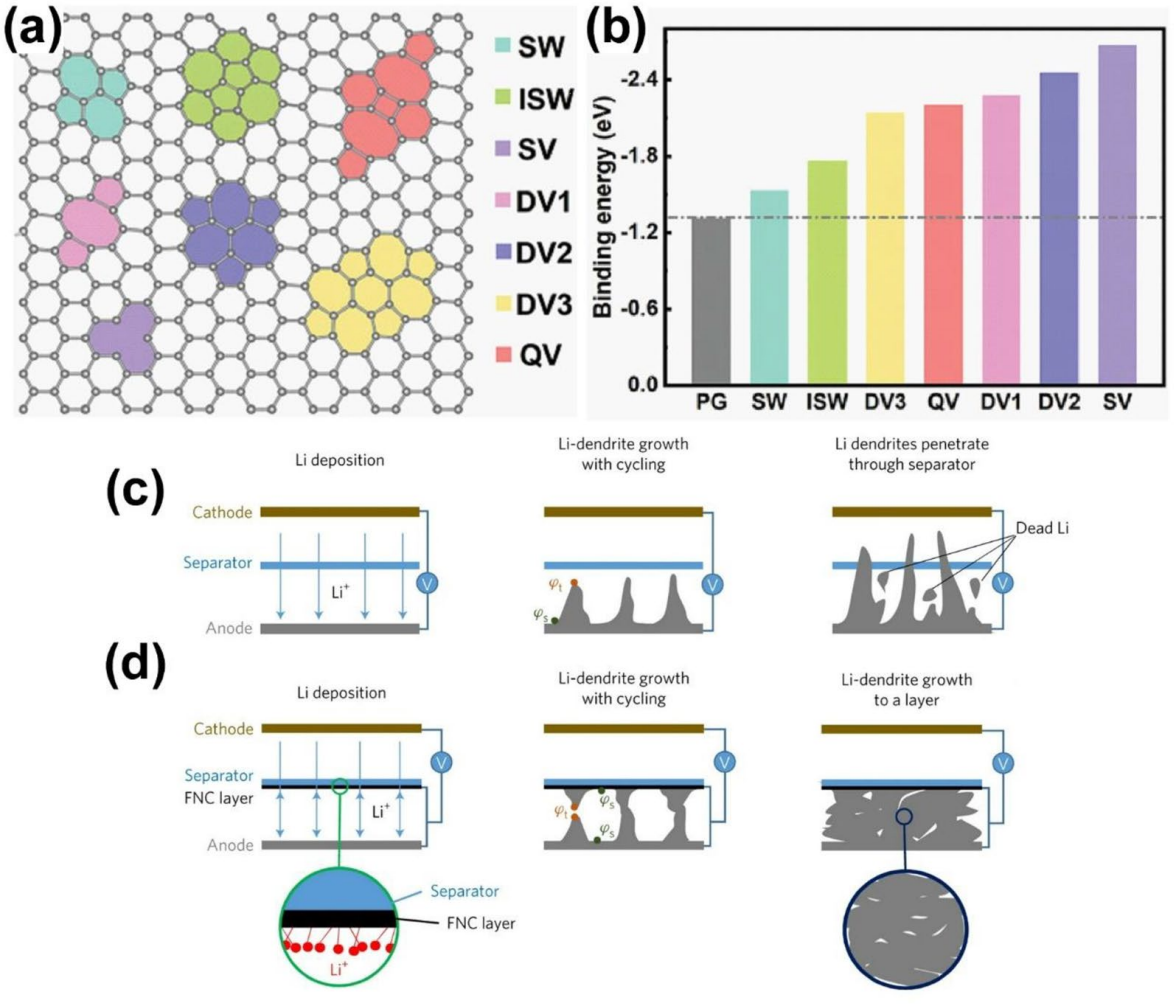
Separator coating for guiding metal nucleation. **a** Models of graphene with defects of Stone–Wales (SW), Inverse Stone–Wales (ISW), single vacancy (SV), three double vacancies (DV1, DV2, DV3) and quadra vacancies (QV). Reprinted with permission from [145]. Copyright 2011, Wiley–VCH. **b** Theoretical calculation results of binding energy of Li on defective graphene. Schematic illustration of lithium dendrite growth in **c** blank and **d** FNC cells. Reprinted with permission from [146]. Copyright 2017, Springer Nature

Dendrite growth always starts from the random metal ion nucleation that happens at the beginning of the metal plating process due to the inhomogeneous metal ion flux. Materials with high metal affinity could effectively regulate the metal nucleation and therefore enable a uniform metal deposition. First principles calculations and experimental study verify the lithiophilicity of carbon frameworks with various heteroatom-doping [144] and defects [145], promoting a uniform Li nucleation for safe metal anode. For example, Zhang’s group discovered that the defects, including single vacancy (SV), double vacancies (DV), quadra vacancies (QV) and Stone–Wales (SW) as illustrated in Fig. 8a, improve the lithiophilicity of graphene compared to the pristine one (Fig. 8b), which helps to break Li bond between Li ion and the electrolyte solvent, and thus reduce the nucleation overpotentials, forming uniform Li deposition. Coating the separator with functionalized nanocarbon (FNC) facing the Li metal electrode surface, as shown in Fig. 8c, d, firstly immobilize the Li^+^ ions within the FNC composite at the lithiophilic sites and then Li grows from the FNC towards Li metal surface with reducing potential difference, $\Delta \varphi$. The Li deposits grow toward each other and eventually merge into dense Li layer between separator and Li anode, instead of dendrites. This strategy greatly improved the cyclability of the Li-metal cell that enabled > 800 cycles with 80% capacity retention [146].

#### MXenes modification on separator

**Fig. 9:**
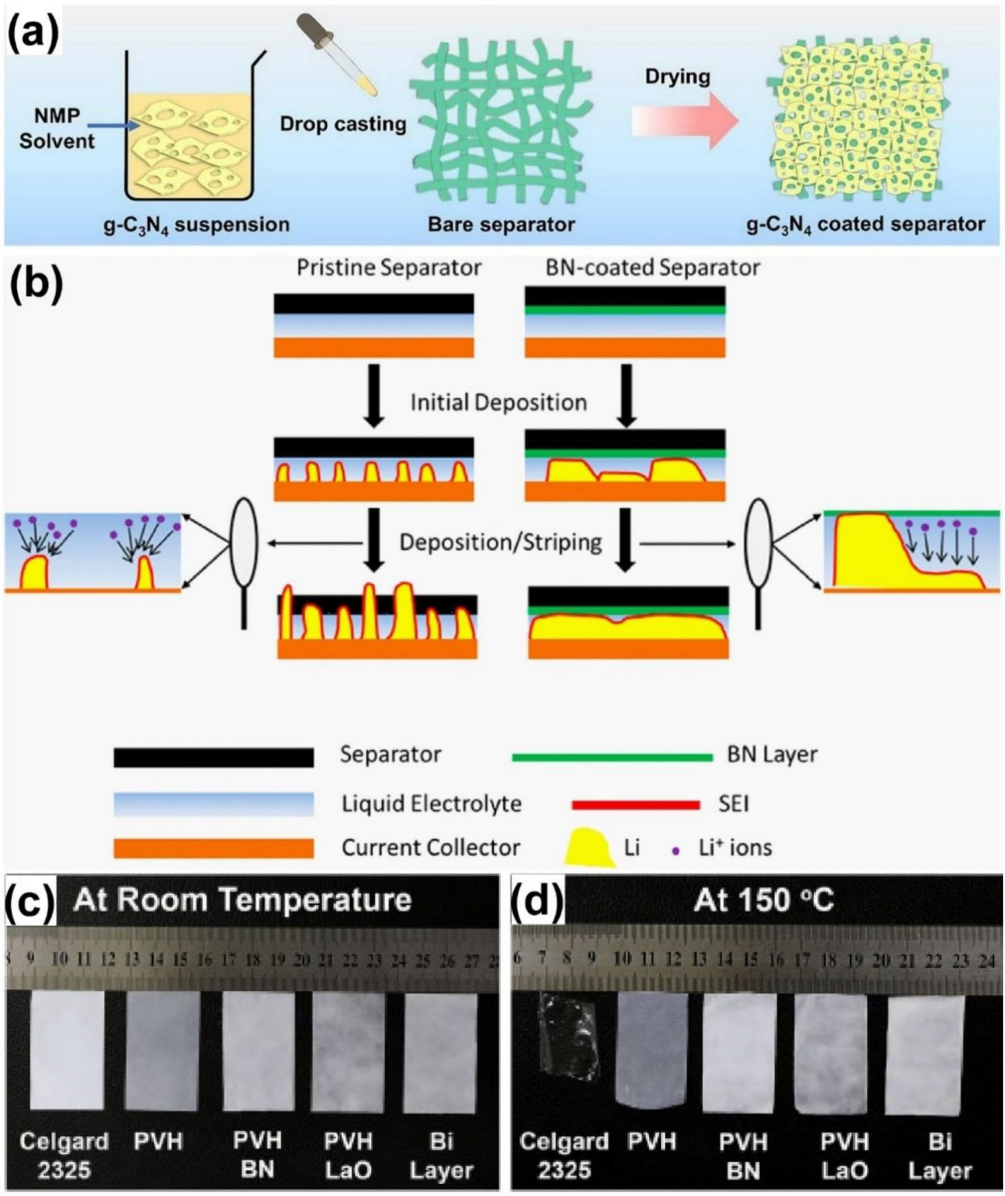
Stable separator coating for enhanced mechanical strength and thermal stability. **a** Graphical illustration of the preparation of g-C_3_N_4_ coated separator using drop casting for safe zinc metal anode. Reprinted with permission from [149]. Copyright 2019, Elsevier. **b** Schematic illustration of inhibiting dendrite process with the thermally conductive BN-coated separator. Reprinted with permission from [151]. Copyright 2015, American Chemical Society. Thermal shrinkage test of separator of Celgard 2325, PVH, PVH-BN, PVH-LaO, and PVH-Lao//PVH-BN (Bilayer) after annealing at **c** room temperatures and **d** 150 °C for 1 h. Reprinted with permission from [153]. Copyright 2019, Elsevier

The unique features of MXenes, including high electrical conductivity and mechanical properties, make it attractive to a variety of applications of energy, catalysis, biomedical fields [147]. To suppress the dendrite formation, layered graphitic carbon nitride (g-C_3_N_4_) was proposed and decorated on the cellulose fiber separator (Fig. 9a), acting as the ion redistributor to homogenize the zinc ion flux, with enhanced mechanical strength of the separator, successfully prevent the Zn dendrite formation, improve the reversibility of Zn metal anode in cycling performance [148]. An et al. [123] fabricated MXene@NiO and coated on glass fiber, which also effectively inhibit the Zn dendrite growth. In addition, 2D nitrogen doped MXene/MOF (N–Ti_3_C_2_/C) nanosheets were developed to prevent the shuttling of polysulfides and suppress the dendrite growth of Li anode in lithium sulfur batteries [149]. The uniformly distributed MOF particles (ZIF-67) on Ti_3_C_2_ act as the spacers to avoid the stacking of MXene 2D geometry, while the nitrogen doping and the active sites from MXene offers sufficient chemical adsorption to the polysulfides and Li^+^ ions as well as homogenize the ion flux, suppressing the dendrite formation in Li anodes.

#### Boron nitride (BN) coating as functional separator

Noted that the internal temperature rise, induced by the uneven electric field and high current density, may cause separator’s thermal shrinkage and lead to short circuit in the battery. Commercial separators, such as, Celgard, are not mechanically strong enough and could be easily punctured by metal dendrites during the metal plating/stripping processes. Therefore, separators with good thermal stability and mechanical strength are the prerequisites for safe metal battery. Owing to the high thermal conductivity (750 W m^−1^ K) and mechanical strength (Young modulus of 80 GPa) of boron nitride (BN) [150]. Hu et al. proposed to decorate BN nanosheets on the separator to homogenize the thermal distribution and lower the risk of dendritic Li growth (Fig. 9b). From the infrared camera, a significant temperature cut from 50 to 34 °C was observed with the BN nanosheets coating, proved the effectiveness of heat dissipation, elongated the cycle life [151]. Furthermore, multifunctional BN-carbon separator was employed to provide mechanical stability and uniform thermal distribution that enhanced the electrochemical performance in the Li–S cell [152]. Moreover, a thermal shrinkage test was performed in Fig. 9c, d, by increasing the temperature from 75 to 150 °C, for 1 h, pristine Celgard 2325 owns 40% of thermal shrinkage, while only 5.2% of shrinkage was observed with the BN modified separator, prove the role of BN in improving the thermal stability of the separator [153].

Similarly, separator modification also is facing the challenges of fabrication complexity and limited compatibility. More importantly, due to the extra coating on the separator, the ionic conductivity is reduced that deteriorate the battery performance. More research efforts should be spent on this topic (Table 2).

**Table 2:** A summary of electrochemical performances for metal batteries using 2D materials on separator engineering for safe metal anode

Material composite	Type of membrane	Coating method	Type of anode	Electrochemical performance
Nitrogen and sulfur codoped graphene (NSG) [142]	PE	Vacuum filtration	Li metal	Couple with LiNi_0.8_Co_0.15_Al_0.05_O_2_, 200 mAh g^−1^ at 1st cycle and 0.5C, 85% retention after 240 cycles
Oxygen and nitrogen doped vertical graphene [143]	Glass fiber	Plasma enhanced chemical vapor deposition (PECVD) + air plasma treatment	Zn metal	Couple with V_2_O_5_, 75% Capacity retention after 1000 cycles
FNC [146]	PP (Celgard 3501)	Slurry coating	Li metal	Couple with LiFePO_4_, 80% retention after 800 cycles, Coulombic efficiency > 97%
N–Ti_3_C_2_/C [149]	PP (Celgard 2400)	Slurry coating	Li metal	Couple with sulfur cathode, 6.3 mAh cm^−2^ with sulfur loading over 10 mg cm^−2^
MXene@NiO [123]	Cellulose fiber	Drop casting	Zn metal	Couple with V_2_O_5_, 275.9 mAh g^−1^ at 0.1 A g^−1^
Graphitic carbon nitride (g-C3N4) [148]	Glass fiber	Slurry coating	Zn metal	
BN nanosheets [151]	PP/PE/PP (Celgard 2325)	Slurry coating	Li metal	Coulombic efficiency: 92% after 100 cycles at 0.5 mA cm^−2^
BN powder [152]	PP	Slurry coating via doctor blading	Li metal	Couple with sulfur cathode, 1036.4 mAh g^−1^ at 1st cycle and 0.5C
PVH-LaO//PVH-BN [153]	PP/PE/PP (Celgard 2325)	Solution coating	Li metal	Couple with LFP, 158 mAh g^−1^ at 0.5 C after 100 cycles

### Electrolyte modifications

Apart from working on the anode surface directly, 2D materials also have been explored as the additives into the liquid or solid electrolyte, which will help to optimize the plating morphology to suppress the dendrite formation [24]. Because of the electron insulating property of graphene-analogous, boron nitride (g-BN)’s lone pair electron [154], it helps to insulate the contact between anode and cathode, and therefore limits the short circuit. The g-BN was reported as the additive into the ionic quasi-liquid solid based electrolyte for LIBs [155]. Toward the deep study of the g-BN, researchers found that the channels between or outside the g-BN also facilitates the smooth transportation of Li ions. Other group also revealed that 2D BN nanoflakes helps to inhibit the dendrite formation since the unprecedented characteristics of high conductivity and Li^+^ transference number [156].

As a family of layered nanosheets structure, MXenes are also emerging as the electrolyte compound in LIBs and SIBs. It has been understood that Ti_3_C_2_T_x_ as a non-aqueous electrolyte promotes the cycling reversibility for sodiation/desodiation process [64]. Owing to the pillaring effect on the ions trapping and swelling effect of the electrolyte penetration, Ti_3_AlC_2_ was synthesized as the part of electrolyte, which contributes to the superb capacity retention [61]. Furthermore, polymer/MXene-based TiO_2_ PVA gel electrolyte presents the excellent ionic conductivity and better mechanical strength which was highly developed in the flexible ZIBs [157]. The cells with polymer/MXene-based TiO_2_ modified-electrolyte delivered over 3000 h lifespan under 0.5 mAcm^−2^.

As for the TMD-based additive, 1T phase MoS_2_ with high electrical conductivity (10–100 S cm^−1^) and intrinsic hydrophilicity was tested as the electrolyte component. The research group demonstrated that exfoliated MoS_2_ is acceptable for the high voltage cycling operation up to 3.5 V, which signs that other metallic phase TMDs should also exhibit favourable electrochemical performance [158].

Critically speaking, the application of 2D materials such as graphene-based additives in the electrolyte of metal batteries is at an early stage. Most of the research focus on the ionic conductivity and mechanical property improvement of the additives in the electrolyte. We consider the further study and discovery of 2D materials-modified electrolyte on the other promising characteristics will be more important.

**Fig. 10:**
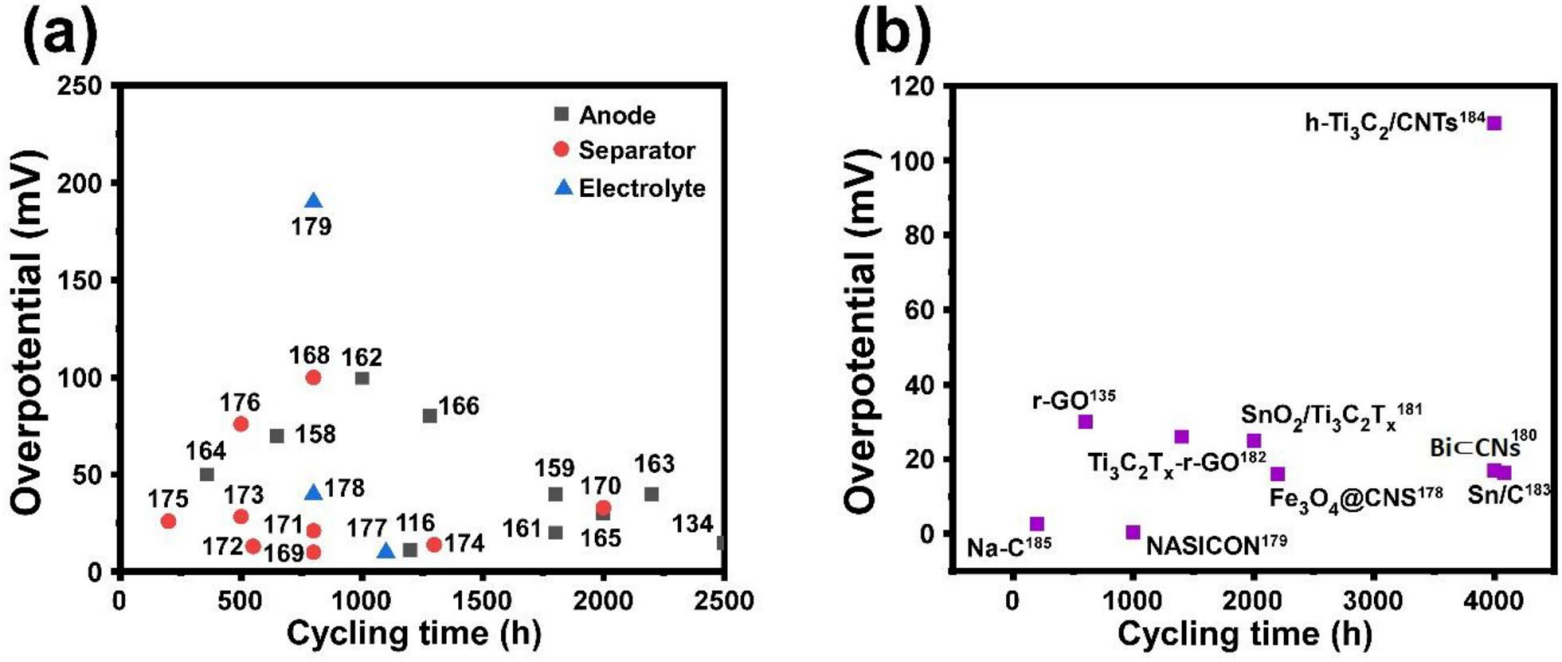
Summarized electrochemical performances of 2D material modifiers for metal anodes. Comparison of the overpotential and cycling time for all 2D material modified anode, separator and electrolyte for **a** Li and **b** Na anodes

Typical 2D materials, including r-GO, MXenes, TMDs and metal nitrides and carbides, modify the cell components of anode, separator, and anodes, successfully supressing the dendrite growth. Figure 10a demonstrates the electrochemical performance comparison between the three different modification methods, anode-based modified, separator typed and electrolyte additives for Li anode, while Fig. 10b compares the electrochemical performances of various 2D materials modifiers for Na anode and the details are given in Table 3.

**Table 3:** Summary of electrochemical performances of 2D materials for modifying anode, separator and electrolyte

2D materials	Cycling time (h)	Overpotential (mV)	References
Li|MXene@Au@Li	650	70	[159]
N-C-SS/Li	1800	40	[160]
MXene (Ti_3_C_2_Cl_x_) layers	1200	11.3	[118]
Au@graphene hybrid aerogel	1800	20	[161]
Graphene/Li_3_N	1000	100	[162]
LiF protected Li/G	2200	40	[163]
F-rich SEI/Li interface on 3D Li-MXene anode	360	50	[164]
LiTiO_2_–Li_3_N–C	2000	30	[165]
SWA-MXene–Li	1280	80	[166]
MoN	2500	15	[136]
Mo_2_C quantum dots (MQDs) anchored N-doped graphene	800	100	[167]
Holey graphene oxide (HGO) on the surface of polyacrylonitrile (PAN) membrane	800	10	[168]
Graphene quantum dots	2000	33	[169]
Mo_2_N@NG	800	21	[170]
Strontium fluoride graphene (SrF_2_-G) sandwich separator	550	13	[171]
V_2_C MXene	500	28.4	[172]
M-HAP@PVHF	1300	13.9	[173]
VS_2_ flakes	200	26	[174]
Ni–VSe_2_/rGO-PP MoS_2_-constructed nanobrushes	500	76	[175]
Graphene oxide (GO) clipped on mesoporous polypyrrole	1100	9	[176]
Glass fiber cloth/poly (ethylene oxide)-MXene	800	40	[177]
Composite ionogel-in-MXene electrolyte (CIME)	800	190	[178]
Fe_3_O_4_@CNS	2200	16	[179]
NASICON	1000	0.25	[180]
Bi ⊂ CNs	4000	17	[181]
SnO_2_/Ti_3_C_2_T_x_	2000	25	[182]
rGO	600	30	[137]
Ti_3_C_2_T_2_–rGO membrane	1400	26	[183]
Sn/C	4080	16.4	[184]
h-Ti_3_C_2_/CNTs	4000	110	[185]
Na-C	200	2.63	[186]

The practical applications of electrolyte modification are limited by the compatibility with other battery components of electrodes, separator, and current collectors. Moreover, the additional of electrolyte components will reduce the ionic conductivity, leading to bad battery performance and poor stability.

## Summary and prospects

As the growing demand for more advanced electricity storage devices, Li, Na and Zn metal batteries have been studied for decades, but are still facing difficulties for commercialization, until resolving the dendrite-driven safety issues. In this review, we summarized the recent advances of 2D materials for safe Li, Na and Zn metal anodes. Owing to the unique properties of 2D materials, including high surface area, mechanical strength, tunable electronic conductivity and abundant active sites, 2D materials were used to construct stable SEI layer, act as 3D hosts for anodes and modify the separator interface and electrolyte, to suppress the dendrite formation by mainly homogenizing the electric field and ion flux, enhancing the mechanical strength and ion conductivity of the separator and SEI layer and guiding the metal ion nucleation direction. Although significant progress has been achieved with the 2D material modifications, several challenges still hinder the practical applications, with our perspectives as stated follows:

Guiding the metal ion nucleation, can manipulate the metal deposition direction at the initial stage of the metal plating process, which is agreed to be the most critical step for inhibiting the dendrite growth. However, limited research has been done on the subsequent metal growth forming metal cluster, which is also important for understanding the underlying reason in suppressing dendrite growth from the nucleated site. For example, most of the theoretical simulations are calculating the metal atom binding strength but not the metal–metal interactions of metal electroplating process. In addition, in-depth effects of 2D materials in suppressing the dendrite growth remains unclear. Currently, very finite analysis methods are used to characterize the metal anode using 2D materials. For instance, to examine the morphology evolution of metal anode structure, SEM and in-situ TEM methods are the dominating characterization strategies. Twisting and mechanic tests with tensile stress are used to check the mechanical stability of the metal anode, while Raman spectroscopy, XRD, XPS, Fourier transform infrared spectroscopy (FTIR) and Brunauer–Emmett Teller (BET) are mainly utilized to examine the anode structure and composition. More advanced characterization techniques, including in situ TEM and in situ Raman spectroscopy, are useful tools to obtain direct evidence and observation for fundamental investigation. Integration with theoretical simulations, we could gain a better understanding of the electrochemical dynamics of metal from thermodynamics point of view.

Moreover, the practical use of 2D materials in metal anodes also faces the challenges that the material preparation method is relatively expensive and complicated, not favorable for industrial operation. Taking the most conventional 2D material of graphene as example, CVD grown graphene has a higher quality in terms of crystallinity with less defects and controllable layer numbers. However, this method requires a high temperature and inert growth environment as well as the time-consuming transfer process, which limits the practical material preparation in large scale. On the other hand, the mechanical exfoliated graphene from graphite owns the disadvantages of uncontrollable layer number and low yields. In contrast, reduction of GO is a more convenient approach to obtain large amounts of graphene in one pot, however, it is hard to control the degree of reduction and products are always bulky. Therefore, more efforts are needed to simplify and reduce the price for large-scale 2D material fabrication.

## References

[1] Reddy MV, Subba Rao GV, Chowdari BV (2013). Metal oxides and oxysalts as anode materials for Li ion batteries. Chem. Rev..

[2] Etacheri V, Marom R, Elazari R, Salitra G, Aurbach D (2011). Challenges in the development of advanced Li-ion batteries: a review. Energy Environ. Sci..

[3] Kim H, Jeong G, Kim YU, Kim JH, Park CM, Sohn HJ (2013). Metallic anodes for next generation secondary batteries. Chem. Soc. Rev..

[4] Scrosati B, Garche J (2010). Lithium batteries: status, prospects and future. J. Power Sources.

[5] Lin D, Liu Y, Cui Y (2017). Reviving the lithium metal anode for high-energy batteries. Nat. Nanotechnol..

[6] Cheng XB, Zhang R, Zhao CZ, Zhang Q (2017). Toward safe lithium metal anode in rechargeable batteries: a review. Chem. Rev..

[7] Sun Y, Liu N, Cui Y (2016). Promises and challenges of nanomaterials for lithium-based rechargeable batteries. Nat. Energy.

[8] Wu F, Lee JT, Zhao E, Zhang B, Yushin G (2016). Graphene-Li_2_S-carbon nanocomposite for lithium–sulfur batteries. ACS Nano.

[9] Manthiram A, Fu Y, Chung SH, Zu C, Su YS (2014). Rechargeable lithium–sulfur batteries. Chem. Rev..

[10] Lee J-S, Tai Kim S, Cao R, Choi N-S, Liu M, Lee KT, Cho J (2011). Metal-air batteries with high energy density: Li-air versus Zn-air. Adv. Energy Mater..

[11] Bruce PG, Freunberger SA, Hardwick LJ, Tarascon JM (2011). Li–O_2_ and Li–S batteries with high energy storage. Nat. Mater..

[12] Fetrow CJ, Carugati C, Zhou X-D, Wei S (2022). Electrochemistry of metal–CO_2_ batteries: opportunities and challenges. Energy Storage Mater..

[13] Xie J, Zhou Z, Wang Y (2019). Metal–CO_2_ batteries at the crossroad to practical energy storage and CO_2_ recycle. Adv. Func. Mater..

[14] Gireaud L, Grugeon S, Laruelle S, Yrieix B, Tarascon JM (2006). Lithium metal stripping/plating mechanisms studies: a metallurgical approach. Electrochem. Commun..

[15] Zhang Y, Qian J, Xu W, Russell SM, Chen X, Nasybulin E, Bhattacharya P, Engelhard MH, Mei D, Cao R, Ding F, Cresce AV, Xu K, Zhang JG (2014). Dendrite-free lithium deposition with self-aligned nanorod structure. Nano Lett..

[16] Qian J, Xu W, Bhattacharya P, Engelhard M, Henderson WA, Zhang Y, Zhang J-G (2015). Dendrite-free Li deposition using trace-amounts of water as an electrolyte additive. Nano Energy.

[17] Lu Y, Tu Z, Archer LA (2014). Stable lithium electrodeposition in liquid and nanoporous solid electrolytes. Nat. Mater..

[18] Liu H, Cheng X-B, Huang J-Q, Yuan H, Lu Y, Yan C, Zhu G-L, Xu R, Zhao C-Z, Hou L-P, He C, Kaskel S, Zhang Q (2020). Controlling dendrite growth in solid-state electrolytes. ACS Energy Lett..

[19] Duan H, Yin YX, Shi Y, Wang PF, Zhang XD, Yang CP, Shi JL, Wen R, Guo YG, Wan LJ (2018). Dendrite-free Li-metal battery enabled by a thin asymmetric solid electrolyte with engineered layers. J. Am. Chem. Soc..

[20] Ren W, Zheng Y, Cui Z, Tao Y, Li B, Wang W (2021). Recent progress of functional separators in dendrite inhibition for lithium metal batteries. Energy Storage Mater..

[21] Hao Z, Zhao Q, Tang J, Zhang Q, Liu J, Jin Y, Wang H (2021). Functional separators towards the suppression of lithium dendrites for rechargeable high-energy batteries. Mater. Horiz..

[22] Zhang Q, Luan J, Tang Y, Ji X, Wang H (2020). Interfacial design of dendrite-free zinc anodes for aqueous zinc-ion batteries. Angew. Chem..

[23] Novoselov KS, Geim AK, Morozov SV, Jiang D, Zhang Y, Dubonos SV, Grigorieva IV, Firsov AA (2004). Electric field effect in atomically thin carbon films. Science.

[24] Zhang C, Wang A, Zhang J, Guan X, Tang W, Luo J (2018). 2D materials for lithium/sodium metal anodes. Adv. Energy Mater..

[25] Peng L, Zhu Y, Chen D, Ruoff RS, Yu G (2016). Two-dimensional materials for beyond-lithium-ion batteries. Adv. Energy Mater..

[26] Zhang C, Pan H, Sun L, Xu F, Ouyang Y, Rosei F (2021). Progress and perspectives of 2D materials as anodes for potassium-ion batteries. Energy Storage Mater..

[27] Ma L, Wu J, Zhu G, Lv Y, Zhang Y, Pang H (2021). Recent advances in two-dimensional materials for alkali metal anodes. J. Mater. Chem. A.

[28] Shi L, Zhao T (2017). Recent advances in inorganic 2D materials and their applications in lithium and sodium batteries. J. Mater. Chem. A.

[29] Wang Y, Xu X, Yin J, Huang G, Guo T, Tian Z, Alsaadi R, Zhu Y, Alshareef HN (2023). MoS_2_-mediated epitaxial plating of Zn metal anodes. Adv. Mater..

[30] Qin G, Liu Y, Liu F, Sun X, Hou L, Liu B, Yuan C (2020). Magnetic field assisted construction of hollow red P nanospheres confined in hierarchical N-doped carbon nanosheets/nanotubes 3D framework for efficient potassium storage. Adv. Energy Mater..

[31] Yao W, Wu S, Zhan L, Wang Y (2019). Two-dimensional porous carbon-coated sandwich-like mesoporous SnO_2_/graphene/mesoporous SnO_2_ nanosheets towards high-rate and long cycle life lithium-ion batteries. Chem. Eng. J..

[32] Zhang C, Mahmood N, Yin H, Liu F, Hou Y (2013). Synthesis of phosphorus-doped graphene and its multifunctional applications for oxygen reduction reaction and lithium ion batteries. Adv. Mater..

[33] Ou X, Yu Y, Wu R, Tyagi A, Zhuang M, Ding Y, Abidi IH, Wu H, Wang F, Luo Z (2018). Shuttle suppression by polymer-sealed graphene-coated polypropylene separator. ACS Appl. Mater Interfaces.

[34] Yan K, Lee HW, Gao T, Zheng G, Yao H, Wang H, Lu Z, Zhou Y, Liang Z, Liu Z, Chu S, Cui Y (2014). Ultrathin two-dimensional atomic crystals as stable interfacial layer for improvement of lithium metal anode. Nano Lett..

[35] Chen K-S, Balla I, Luu NS, Hersam MC (2017). Emerging opportunities for two-dimensional materials in lithium-ion batteries. ACS Energy Lett..

[36] Garg S, Parmar AS, Rosy R (2023). Hexagonal boron nitride as anode for sodium ion battery—a reality check!. J. Electrochem. Soc..

[37] Wong H, Ou X, Zhuang M, Liu Z, Hossain MD, Cai Y, Liu H, Lee H, Wang CZ, Luo Z (2019). Selenium edge as a selective anchoring site for lithium–sulfur batteries with MoSe_2_/graphene-based cathodes. ACS Appl. Mater. Interfaces.

[38] Zhang S, Wang G, Jin J, Zhang L, Wen Z, Yang J (2018). Robust and conductive red MoSe_2_ for stable and fast lithium storage. ACS Nano.

[39] Cheng Z, Xiao Z, Pan H, Wang S, Wang R (2018). Elastic sandwich-type rGO-VS_2_/S composites with high tap density: structural and chemical cooperativity enabling lithium–sulfur batteries with high energy density. Adv. Energy Mater..

[40] Zhou J, Lin S, Huang Y, Tong P, Zhao B, Zhu X, Sun Y (2019). Synthesis and lithium ion storage performance of two-dimensional V_4_C_3_ MXene. Chem. Eng. J..

[41] Liu Y, Sun Z, Sun X, Lin Y, Tan K, Sun J, Liang L, Hou L, Yuan C (2020). Construction of hierarchical nanotubes assembled from ultrathin V_3_S_4_@C nanosheets towards alkali-ion batteries with ion-dependent electrochemical mechanisms. Angew. Chem..

[42] Simon P (2017). Two-dimensional MXene with controlled interlayer spacing for electrochemical energy storage. ACS Nano.

[43] Che Z, Li Y, Chen K, Wei M (2016). Hierarchical MoS_2_@RGO nanosheets for high performance sodium storage. J. Power Sources.

[44] Xie X, Ao Z, Su D, Zhang J, Wang G (2015). MoS_2_/graphene composite anodes with enhanced performance for sodium-ion batteries: the role of the two-dimensional heterointerface. Adv. Func. Mater..

[45] Geng X, Jiao Y, Han Y, Mukhopadhyay A, Yang L, Zhu H (2017). Freestanding metallic 1T MoS_2_ with dual ion diffusion paths as high rate anode for sodium-ion batteries. Adv. Func. Mater..

[46] Huang X, Zeng Z, Zhang H (2013). Metal dichalcogenide nanosheets: preparation, properties and applications. Chem. Soc. Rev..

[47] Zhi C, Bando Y, Tang C, Kuwahara H, Golberg D (2009). Large-scale fabrication of boron nitride nanosheets and their utilization in polymeric composites with improved thermal and mechanical properties. Adv. Mater..

[48] Wu ZS, Ren W, Xu L, Li F, Cheng HM (2011). Doped graphene sheets as anode materials with superhigh rate and large capacity for lithium ion batteries. ACS Nano.

[49] Mazar Atabaki M, Kovacevic R (2013). Graphene composites as anode materials in lithium-ion batteries. Electron. Mater. Lett..

[50] Gong Y, Yang S, Liu Z, Ma L, Vajtai R, Ajayan PM (2013). Graphene-network-backboned architectures for high-performance lithium storage. Adv. Mater..

[51] Shen B, Zhang TW, Yin YC, Zhu ZX, Lu LL, Ma C, Zhou F, Yao HB (2019). Chemically exfoliated boron nitride nanosheets form robust interfacial layers for stable solid-state Li metal batteries. Chem. Commun. (Camb).

[52] Cheng Q, Li A, Li N, Li S, Zangiabadi A, Li T-D, Huang W, Li AC, Jin T, Song Q, Xu W, Ni N, Zhai H, Dontigny M, Zaghib K, Chuan X, Su D, Yan K, Yang Y (2019). Stabilizing solid electrolyte-anode interface in Li-metal batteries by boron nitride-based nanocomposite coating. Joule.

[53] Su D, Dou S, Wang G (2015). Ultrathin MoS_2_ nanosheets as anode materials for sodium-ion batteries with superior performance. Adv. Energy Mater..

[54] Fan X-L, Yang Y, Xiao P, Lau W-M (2014). Site-specific catalytic activity in exfoliated MoS_2_ single-layer polytypes for hydrogen evolution: basal plane and edges. J. Mater. Chem. A.

[55] Chao D, Zhu C, Yang P, Xia X, Liu J, Wang J, Fan X, Savilov SV, Lin J, Fan HJ, Shen ZX (2016). Array of nanosheets render ultrafast and high-capacity Na-ion storage by tunable pseudocapacitance. Nat. Commun..

[56] Geng X, Zhang Y, Han Y, Li J, Yang L, Benamara M, Chen L, Zhu H (2017). Two-dimensional water-coupled metallic MoS_2_ with nanochannels for ultrafast supercapacitors. Nano Lett..

[57] Jia G, Chao D, Tiep NH, Zhang Z, Fan HJ (2018). Intercalation Na-ion storage in two-dimensional MoS_2-x_Se_x_ and capacity enhancement by selenium substitution. Energy Storage Mater..

[58] Oh HG, Yang SH, Kang YC, Park SK (2021). N-dopedcarbon-coated CoSe_2_ nanocrystals anchored on two-dimensional MXene nanosheets for efficient electrochemical sodium- and potassium-ion storage. Int. J. Energy Res..

[59] Yang SH, Park SK, Kang YC (2021). Metal-organic frameworks derived FeSe_2_@C nanorods interconnected by N-doped graphene nanosheets as advanced anode materials for Na-ion batteries. Int. J. Energy Res..

[60] Wu Y, Nie P, Wang J, Dou H, Zhang X (2017). Few-layer MXenes delaminated via high-energy mechanical milling for enhanced sodium-ion batteries performance. ACS Appl. Mater. Interfaces.

[61] Kajiyama S, Szabova L, Sodeyama K, Iinuma H, Morita R, Gotoh K, Tateyama Y, Okubo M, Yamada A (2016). Sodium-ion intercalation mechanism in MXene nanosheets. ACS Nano.

[62] Sun D, Wang M, Li Z, Fan G, Fan L-Z, Zhou A (2014). Two-dimensional Ti_3_C_2_ as anode material for Li-ion batteries. Electrochem. Commun..

[63] Yang E, Ji H, Kim J, Kim H, Jung Y (2015). Exploring the possibilities of two-dimensional transition metal carbides as anode materials for sodium batteries. Phys. Chem. Chem. Phys..

[64] Naguib M, Kurtoglu M, Presser V, Lu J, Niu J, Heon M, Hultman L, Gogotsi Y, Barsoum MW (2011). Two-dimensional nanocrystals produced by exfoliation of Ti_3_AlC_2_. Adv. Mater..

[65] Yu Y-X (2016). Prediction of mobility, enhanced storage capacity, and volume change during sodiation on interlayer-expanded functionalized Ti_3_C_2_ MXene anode materials for sodium-ion batteries. J. Phys. Chem. C.

[66] Zhao S, Kang W, Xue J (2015). MXene nanoribbons. J. Mater. Chem. C.

[67] Shpigel N, Lukatskaya MR, Sigalov S, Ren CE, Nayak P, Levi MD, Daikhin L, Aurbach D, Gogotsi Y (2017). In situ monitoring of gravimetric and viscoelastic changes in 2D intercalation electrodes. ACS Energy Lett..

[68] Wang X, Kajiyama S, Iinuma H, Hosono E, Oro S, Moriguchi I, Okubo M, Yamada A (2015). Pseudocapacitance of MXene nanosheets for high-power sodium-ion hybrid capacitors. Nat. Commun..

[69] Sun Y, Du C, Zhou C, Zhu X, Chen J (2019). Analysis of load-induced top-down cracking initiation in asphalt pavements using a two-dimensional microstructure-based multiscale finite element method. Eng. Fract. Mech..

[70] Lai WH, Wang YX, Wang JZ, Chou SL, Dou SX (2020). Manipulating 2D few-layer metal sulfides as anode towards enhanced sodium-ion batteries. Batteries Supercaps.

[71] Paiva AC, Simões Oliveira D, Hantao LW (2019). A bottom-up approach for data mining in bioaromatization of beers using flow-modulated comprehensive two-dimensional gas chromatography/mass spectrometry. Separations.

[72] Zeng T, Yang H, Wang H, Chen G (2020). Acepentalene membrane sheet: a metallic two-dimensional carbon allotrope with high carrier mobility for lithium ion battery anodes. J. Phys. Chem. C.

[73] Wang Y, Fu XW, Zheng M, Zhong WH, Cao GZ (2019). Strategies for building robust traffic networks in advanced energy storage devices: a focus on composite electrodes. Adv. Mater..

[74] Chen J, Duan M, Chen G (2012). Continuous mechanical exfoliation of graphene sheets via three-roll mill. J. Mater. Chem..

[75] Samorì P, Feng X, Palermo V (2020). Introduction to ‘chemistry of 2D materials: graphene and beyond’. Nanoscale.

[76] Abdillah OB, Floweri O, Irham MA, Aimon AH, Ogi T, Iskandar F (2022). Structural modulation of exfoliated graphene via a facile postultrasonication treatment toward enhanced electrochemical properties of supercapacitor electrode. Energy Fuels.

[77] Berktas I, Hezarkhani M, Haghighi Poudeh L, Saner Okan B (2020). Recent developments in the synthesis of graphene and graphene-like structures from waste sources by recycling and upcycling technologies: a review. Graphene Technol..

[78] Hu M, Zhou Y, Nie W, Chen P (2018). Functionalized graphene nanosheets with fewer defects prepared via sodium alginate assisted direct exfoliation of graphite in aqueous media for lithium-ion batteries. ACS Appl. Nano Mater..

[79] Garcia-Dali S, Paredes JI, Munuera JM, Villar-Rodil S, Adawy A, Martinez-Alonso A, Tascón JM (2019). Aqueous cathodic exfoliation strategy toward solution-processable and phase-preserved MoS_2_ nanosheets for energy storage and catalytic applications. ACS Appl. Mater. Interfaces.

[80] Hernandez Y, Nicolosi V, Lotya M, Blighe FM, Sun Z, De S, McGovern IT, Holland B, Byrne M, Gun'Ko YK (2008). High-yield production of graphene by liquid-phase exfoliation of graphite. Nat. Nanotechnol..

[81] Guardia L, Fernández-Merino M, Paredes J, Solís-Fernández P, Villar-Rodil S, Martínez-Alonso A, Tascón J (2011). High-throughput production of pristine graphene in an aqueous dispersion assisted by non-ionic surfactants. Carbon.

[82] Yi M, Shen Z, Ma S, Zhang X (2012). A mixed-solvent strategy for facile and green preparation of graphene by liquid-phase exfoliation of graphite. J. Nanopart. Res..

[83] Lotya M, Hernandez Y, King PJ, Smith RJ, Nicolosi V, Karlsson LS, Blighe FM, De S, Wang Z, McGovern I (2009). Liquid phase production of graphene by exfoliation of graphite in surfactant/water solutions. J. Am. Chem. Soc..

[84] Lotya M, King PJ, Khan U, De S, Coleman JN (2010). High-concentration, surfactant-stabilized graphene dispersions. ACS Nano.

[85] Ciesielski A, Samorì P (2014). Graphene via sonication assisted liquid-phase exfoliation. Chem. Soc. Rev..

[86] Cravotto G, Cintas P (2010). Sonication-assisted fabrication and post-synthetic modifications of graphene-like materials. Chem. Eur. J..

[87] Liu B, Luo T, Mu G, Wang X, Chen D, Shen G (2013). Rechargeable Mg-ion batteries based on WSe_2_ nanowire cathodes. ACS Nano.

[88] Seo D-B, Yoo S, Dongquoc V, Trung TN, Kim E-T (2021). Facile synthesis and efficient photoelectrochemical reaction of WO_3_/WS_2_ core@ shell nanorods utilizing WO_3_·0.33H_2_O phase. J Alloys Compd..

[89] Kang T, Tang TW, Pan B, Liu H, Zhang K, Luo Z (2022). Strategies for controlled growth of transition metal dichalcogenides by chemical vapor deposition for integrated electronics. ACS Mater. Au.

[90] You J, Hossain MD, Luo Z (2018). Synthesis of 2D transition metal dichalcogenides by chemical vapor deposition with controlled layer number and morphology. Nano Converg..

[91] Zhang L, Dong J, Ding F (2021). Strategies, status, and challenges in wafer scale single crystalline two-dimensional materials synthesis. Chem. Rev..

[92] Zhou L, Yan S, Lin Z, Shi Y (2016). In situ reduction of WS_2_ nanosheets for WS_2_/reduced graphene oxide composite with superior Li-ion storage. Mater. Chem. Phys..

[93] Song Y, Liao J, Chen C, Yang J, Chen J, Gong F, Wang S, Xu Z, Wu M (2019). Controllable morphologies and electrochemical performances of self-assembled nano-honeycomb WS_2_ anodes modified by graphene doping for lithium and sodium ion batteries. Carbon.

[94] Zhou R, Wang H, Chang J, Yu C, Dai H, Chen Q, Zhou J, Yu H, Sun G, Huang W (2021). Ammonium intercalation induced expanded 1T-rich molybdenum diselenides for improved lithium ion storage. ACS Appl. Mater. Interfaces.

[95] Marri SR, Ratha S, Rout CS, Behera J (2017). 3D cuboidal vanadium diselenide embedded reduced graphene oxide hybrid structures with enhanced supercapacitor properties. Chem. Commun..

[96] Wang F, Li Y, Shifa TA, Liu K, Wang F, Wang Z, Xu P, Wang Q, He J (2016). Selenium-enriched nickel selenide nanosheets as a robust electrocatalyst for hydrogen generation. Angew. Chem. Int. Ed..

[97] Bao S-J, Li CM, Guo C-X, Qiao Y (2008). Biomolecule-assisted synthesis of cobalt sulfide nanowires for application in supercapacitors. J. Power Sources.

[98] Li J, Zhou X, Xia Z, Zhang Z, Li J, Ma Y, Qu Y (2015). Facile synthesis of CoX (X=S, P) as an efficient electrocatalyst for hydrogen evolution reaction. J. Mater. Chem. A.

[99] Peng S, Li L, Mhaisalkar SG, Srinivasan M, Ramakrishna S, Yan Q (2014). Hollow nanospheres constructed by CoS_2_ nanosheets with a nitrogen-doped-carbon coating for energy-storage and photocatalysis. ChemSusChem.

[100] Fang X, Wang Z, Kang S, Zhao L, Jiang Z, Dong M (2020). Hexagonal CoSe_2_ nanosheets stabilized by nitrogen-doped reduced graphene oxide for efficient hydrogen evolution reaction. Int. J. Hydrogen Energy.

[101] Bhardwaj T, Antic A, Pavan B, Barone V, Fahlman BD (2010). Enhanced electrochemical lithium storage by graphene nanoribbons. J. Am. Chem. Soc..

[102] Ito Y, Christodoulou C, Nardi MV, Koch N, Sachdev H, Mullen K (2014). Chemical vapor deposition of N-doped graphene and carbon films: the role of precursors and gas phase. ACS Nano.

[103] Yun YS, Le V-D, Kim H, Chang S-J, Baek SJ, Park S, Kim BH, Kim Y-H, Kang K, Jin H-J (2014). Effects of sulfur doping on graphene-based nanosheets for use as anode materials in lithium-ion batteries. J. Power Sources.

[104] Terrones M, Botello-Méndez AR, Campos-Delgado J, López-Urías F, Vega-Cantú YI, Rodríguez-Macías FJ, Elías AL, Muñoz-Sandoval E, Cano-Márquez AG, Charlier J-C (2010). Graphene and graphite nanoribbons: morphology, properties, synthesis, defects and applications. Nano Today.

[105] Bai M, Xie K, Yuan K, Zhang K, Li N, Shen C, Lai Y, Vajtai R, Ajayan P, Wei B (2018). A scalable approach to dendrite-free lithium anodes via spontaneous reduction of spray-coated graphene oxide layers. Adv. Mater..

[106] Murugan AV, Muraliganth T, Manthiram A (2009). Rapid, facile microwave-solvothermal synthesis of graphene nanosheets and their polyaniline nanocomposites for energy storage. Chem. Mater..

[107] Suragtkhuu S, Sunderiya S, Myagmarsereejid P, Purevdorj S, Bati ASR, Bold B, Zhong YL, Davaasambuu S, Batmunkh M (2023). Graphene-like monoelemental 2D materials for perovskite solar cells. Adv. Energy Mater..

[108] Bi J, Du Z, Sun J, Liu Y, Wang K, Du H, Ai W, Huang W (2023). On the road to the frontiers of lithium-ion batteries: a review and outlook of graphene anodes. Adv. Mater..

[109] Wang L, Deng J, Deng J, Fei Y, Fang Y, Hu YH (2020). Ultra-fast and ultra-long-life Li ion batteries with 3D surface-porous graphene anodes synthesized from CO_2_. J. Mater. Chem. A.

[110] Jang J-H, Rangappa D, Kwon Y-U, Honma I (2011). Direct preparation of 1-PSA modified graphene nanosheets by supercritical fluidic exfoliation and its electrochemical properties. J. Mater. Chem..

[111] Lou D, Chen S, Langrud S, Razzaq AA, Mao M, Younes H, Xing W, Lin T, Hong H (2022). Scalable fabrication of Si–graphene composite as anode for Li-ion batteries. Appl. Sci..

[112] Son IH, Hwan Park J, Kwon S, Park S, Rummeli MH, Bachmatiuk A, Song HJ, Ku J, Choi JW, Choi JM, Doo SG, Chang H (2015). Silicon carbide-free graphene growth on silicon for lithium-ion battery with high volumetric energy density. Nat. Commun..

[113] Yao F, Gunes F, Ta HQ, Lee SM, Chae SJ, Sheem KY, Cojocaru CS, Xie SS, Lee YH (2012). Diffusion mechanism of lithium ion through basal plane of layered graphene. J. Am. Chem. Soc..

[114] Wu Z-S, Xue L, Ren W, Li F, Wen L, Cheng H-M (2012). A LiF nanoparticle-modified graphene electrode for high-power and high-energy lithium ion batteries. Adv. Func. Mater..

[115] Liu XH, Wang JW, Liu Y, Zheng H, Kushima A, Huang S, Zhu T, Mao SX, Li J, Zhang S, Lu W, Tour JM, Huang JY (2012). In situ transmission electron microscopy of electrochemical lithiation, delithiation and deformation of individual graphene nanoribbons. Carbon.

[116] Xu M, Li Y, Ihsan-Ul-Haq M, Mubarak N, Liu Z, Wu J, Luo Z, Kim JK (2022). NaF-rich solid electrolyte interphase for dendrite-free sodium metal batteries. Energy Storage Mater..

[117] Zheng J, Zhao Q, Tang T, Yin J, Quilty CD, Renderos GD, Liu X, Deng Y, Wang L, Bock DC, Jaye C, Zhang D, Takeuchi ES, Takeuchi KJ, Marschilok AC, Archer LA (2019). Reversible epitaxial electrodeposition of metals in battery anodes. Science.

[118] Gu J, Zhu Q, Shi Y, Chen H, Zhang D, Du Z, Yang S (2020). Single zinc atoms immobilized on MXene (Ti_3_C_2_Cl_x_) layers toward dendrite-free lithium metal anodes. ACS Nano.

[119] Luo J, Lu X, Matios E, Wang C, Wang H, Zhang Y, Hu X, Li W (2020). Tunable MXene-derived 1D/2D hybrid nanoarchitectures as a stable matrix for dendrite-free and ultrahigh capacity sodium metal anode. Nano Lett..

[120] Zhang N, Huang S, Yuan Z, Zhu J, Zhao Z, Niu Z (2021). Direct self-assembly of MXene on Zn anodes for dendrite-free aqueous zinc-ion batteries. Angew. Chem..

[121] Zhang D, Wang S, Li B, Gong Y, Yang S (2019). Horizontal growth of lithium on parallelly aligned MXene layers towards dendrite-free metallic lithium anodes. Adv Mater.

[122] Chen X, Shang M, Niu J (2020). Inter-layer-calated thin Li metal electrode with improved battery capacity retention and dendrite suppression. Nano Lett..

[123] An Y, Tian Y, Man Q, Shen H, Liu C, Qian Y, Xiong S, Feng J, Qian Y (2022). Highly reversible Zn metal anodes enabled by freestanding, lightweight, and zincophilic MXene/nanoporous oxide heterostructure engineered separator for flexible Zn-MnO_2_ batteries. ACS Nano.

[124] Tian Y, An Y, Liu C, Xiong S, Feng J, Qian Y (2021). Reversible zinc-based anodes enabled by zincophilic antimony engineered MXene for stable and dendrite-free aqueous zinc batteries. Energy Storage Mater..

[125] Yang S-Y, Shi D-R, Wang T, Yue X-Y, Zheng L, Zhang Q-H, Gu L, Yang X-Q, Shadike Z, Li H, Fu Z-W (2020). High-rate cathode CrSSe based on anion reactions for lithium-ion batteries. J. Mater. Chem. A.

[126] Cha E, Patel MD, Park J, Hwang J, Prasad V, Cho K, Choi W (2018). 2D MoS_2_ as an efficient protective layer for lithium metal anodes in high-performance Li–S batteries. Nat. Nanotechnol..

[127] Xu S, Gao X, Hua Y, Neville A, Wang Y, Zhang K (2020). Rapid deposition of WS_2_ platelet thin films as additive-free anode for sodium ion batteries with superior volumetric capacity. Energy Storage Mater..

[128] Cui Y, Xiao K, Bedford NM, Lu X, Yun J, Amal R, Wang DW (2019). Refilling nitrogen to oxygen vacancies in ultrafine tungsten oxide clusters for superior lithium storage. Adv. Energy Mater..

[129] Zeng X, Ding Z, Ma C, Wu L, Liu J, Chen L, Ivey DG, Wei W (2016). Hierarchical nanocomposite of hollow N-doped carbon spheres decorated with ultrathin WS_2_ nanosheets for high-performance lithium-ion battery anode. ACS Appl. Mater. Interfaces.

[130] Che G, Jirage KB, Fisher ER, Martin CR, Yoneyama H (2019). Chemical-vapor deposition-based template synthesis of microtubular TiS_2_ battery electrodes. J. Electrochem. Soc..

[131] Li Y, Wong H, Wang J, Peng W, Shen Y, Xu M, An Q, Kim JK, Yuan B, Goddard WA, Luo Z (2022). Deposition of horizontally stacked Zn Crystals on single layer 1T-VSe_2_ for dendrite-free Zn metal anodes. Adv. Energy Mater..

[132] Cao Z, Zhang Y, Cui Y, Gu J, Du Z, Shi Y, Shen K, Chen H, Li B, Yang S (2021). Harnessing the unique features of 2D materials toward dendrite-free metal anodes. Energy Environ. Mater..

[133] Yang SH, Lee YJ, Kang H, Park SK, Kang YC (2021). Carbon-coated three-dimensional MXene/iron selenide ball with core-shell structure for high-performance potassium-ion batteries. Nanomicro Lett..

[134] Lin D, Liu Y, Liang Z, Lee HW, Sun J, Wang H, Yan K, Xie J, Cui Y (2016). Layered reduced graphene oxide with nanoscale interlayer gaps as a stable host for lithium metal anodes. Nat. Nanotechnol..

[135] Cao Z, Zhu Q, Wang S, Zhang D, Chen H, Du Z, Li B, Yang S (2019). Perpendicular MXene arrays with periodic interspaces toward dendrite-free lithium metal anodes with high-rate capabilities. Adv. Func. Mater..

[136] Gao D, Deng S, Li X, Zhang Y, Lv T, He Y, Mao W, Yang H, Zhang J, Chu PK, Huo K (2023). Lithiophilic and conductive framework of 2D MoN nanosheets enabling planar lithium plating for dendrite-free and minimum-volume-change lithium metal anodes. Chem. Eng. J..

[137] Wang A, Hu X, Tang H, Zhang C, Liu S, Yang YW, Yang QH, Luo J (2017). Processable and moldable sodium-metal anodes. Angew. Chem..

[138] Pan Y, Chou S, Liu HK, Dou SX (2017). Functional membrane separators for next-generation high-energy rechargeable batteries. Natl. Sci. Rev..

[139] Hou Z, Gao Y, Tan H, Zhang B (2021). Realizing high-power and high-capacity zinc/sodium metal anodes through interfacial chemistry regulation. Nat. Commun..

[140] Zhao CZ, Chen PY, Zhang R, Chen X, Li BQ, Zhang XQ, Cheng XB, Zhang Q (2018). An ion redistributor for dendrite-free lithium metal anodes. Sci. Adv..

[141] Qin Y, Liu P, Zhang Q, Wang Q, Sun D, Tang Y, Ren Y, Wang H (2020). Advanced filter membrane separator for aqueous zinc-ion batteries. Small.

[142] Shin WK, Kannan AG, Kim DW (2015). Effective suppression of dendritic lithium growth using an ultrathin coating of nitrogen and sulfur codoped graphene nanosheets on polymer separator for lithium metal batteries. ACS Appl. Mater. Interfaces.

[143] Li C, Sun Z, Yang T, Yu L, Wei N, Tian Z, Cai J, Lv J, Shao Y, Rummeli MH, Sun J, Liu Z (2020). Directly grown vertical graphene carpets as Janus separators toward stabilized Zn metal anodes. Adv. Mater..

[144] Chen X, Chen XR, Hou TZ, Li BQ, Cheng XB, Zhang R, Zhang Q (2019). Lithiophilicity chemistry of heteroatom-doped carbon to guide uniform lithium nucleation in lithium metal anodes. Sci. Adv..

[145] Ma XX, Chen X, Bai YK, Shen X, Zhang R, Zhang Q (2021). The defect chemistry of carbon frameworks for regulating the lithium nucleation and growth behaviors in lithium metal anodes. Small.

[146] Liu Y, Liu Q, Xin L, Liu Y, Yang F, Stach EA, Xie J (2017). Making Li-metal electrodes rechargeable by controlling the dendrite growth direction. Nat. Energy.

[147] Gogotsi Y, Anasori B (2019). The rise of MXenes. ACS Nano.

[148] Yang Y, Chen T, Yu B, Zhu M, Meng F, Shi W, Zhang M, Qi Z, Zeng K, Xue J (2022). Manipulating Zn-ion flux by two-dimensional porous g-C3N4 nanosheets for dendrite-free zinc metal anode. Chem. Eng. J..

[149] Jiang G, Zheng N, Chen X, Ding G, Li Y, Sun F, Li Y (2019). In-situ decoration of MOF-derived carbon on nitrogen-doped ultrathin MXene nanosheets to multifunctionalize separators for stable Li–S batteries. Chem. Eng. J..

[150] Su M, Huang G, Wang S, Wang Y, Wang H (2021). High safety separators for rechargeable lithium batteries. Sci. China Chem..

[151] Luo W, Zhou L, Fu K, Yang Z, Wan J, Manno M, Yao Y, Zhu H, Yang B, Hu L (2015). A thermally conductive separator for stable Li metal anodes. Nano Lett..

[152] Kim PJH, Seo J, Fu K, Choi J, Liu Z, Kwon J, Hu L, Paik U (2017). Synergistic protective effect of a BN-carbon separator for highly stable lithium sulfur batteries. NPG Asia Mater..

[153] Waqas M, Ali S, Chen D, Boateng B, Han Y, Zhang M, Han J, Goodenough JB, He W (2019). A robust bi-layer separator with Lewis acid–base interaction for high-rate capacity lithium-ion batteries. Compos. B Eng..

[154] Weng Q, Wang X, Wang X, Bando Y, Golberg D (2016). Functionalized hexagonal boron nitride nanomaterials: emerging properties and applications. Chem Soc Rev.

[155] Li M, Zhu W, Zhang P, Chao Y, He Q, Yang B, Li H, Borisevich A, Dai S (2016). Graphene-analogues boron nitride nanosheets confining ionic liquids: a high-performance quasi-liquid solid electrolyte. Small.

[156] Shim J, Kim HJ, Kim BG, Kim YS, Kim D-G, Lee J-C (2017). 2D boron nitride nanoflakes as a multifunctional additive in gel polymer electrolytes for safe, long cycle life and high rate lithium metal batteries. Energy Environ. Sci..

[157] Liu C, Tian Y, An Y, Yang Q, Xiong S, Feng J, Qian Y (2022). Robust and flexible polymer/MXene-derived two dimensional TiO_2_ hybrid gel electrolyte for dendrite-free solid-state zinc-ion batteries. Chem. Eng. J..

[158] Acerce M, Voiry D, Chhowalla M (2015). Metallic 1T phase MoS_2_ nanosheets as supercapacitor electrode materials. Nat. Nanotechnol..

[159] Qian Y, Wei C, Tian Y, Xi B, Xiong S, Feng J, Qian Y (2021). Constructing ultrafine lithiophilic layer on MXene paper by sputtering for stable and flexible 3D lithium metal anode. Chem. Eng. J..

[160] Li Y, Min Y, Liang J, Liu Z, Yuan B, Xu L, Luo Z, Zhu M (2021). Lithiophilic diffusion barrier layer on stainless steel mesh for dendrite suppression and stable lithium metal anode. Appl. Mater. Today.

[161] Yang T, Li L, Wu F, Chen R (2020). A soft lithiophilic graphene aerogel for stable lithium metal anode. Adv. Func. Mater..

[162] Wan M, Duan X, Cui H, Du J, Fu L, Chen Z, Lu Z, Li G, Li Y, Mao E, Wang L, Sun Y (2022). Stabilized Li metal anode with robust C-Li_3_N interphase for high energy density batteries. Energy Storage Mater..

[163] Liu Z, He B, Zhang Z, Deng W, Dong D, Xia S, Zhou X, Liu Z (2022). Lithium/graphene composite anode with 3D structural LiF protection layer for high-performance lithium metal batteries. ACS Appl. Mater. Interfaces.

[164] Qian Y, Zhang K, Tan L, An Y, Xi B, Xiong S, Feng J, Qian Y (2022). Highly reversible and safe lithium metal batteries enabled by non-flammable all-fluorinated carbonate electrolyte conjugated with 3D flexible MXene-based lithium anode. Chem. Eng. J..

[165] Wang J, Yang M, Zou G, Liu D, Peng Q (2021). Lithiation MXene derivative skeletons for wide-temperature lithium metal anodes. Adv. Func. Mater..

[166] Gu J, Chen H, Shi Y, Cao Z, Du Z, Li B, Yang S (2022). Eliminating lightning-rod effect of lithium anodes via sine-wave analogous MXene layers. Adv. Energy Mater..

[167] Yu B, Chen D, Wang Z, Qi F, Zhang X, Wang X, Hu Y, Wang B, Zhang W, Chen Y, He J, He W (2020). Mo_2_C quantum dots@graphene functionalized separator toward high-current-density lithium metal anodes for ultrastable Li–S batteries. Chem. Eng. J..

[168] Chen Y, Li J, Ju Y, Cheng R, Zhai Y, Sheng J, Liu H, Li L (2022). Regulating Li-ion flux distribution via holey graphene oxide functionalized separator for dendrite-inhibited lithium metal battery. Appl. Surf. Sci..

[169] Senthil C, Kim SG, Kim SS, Hahm MG, Jung HY (2022). Robust, ultrasmooth fluorinated lithium metal interphase feasible via lithiophilic graphene quantum dots for dendrite-less batteries. Small.

[170] Ma F, Srinivas K, Zhang X, Zhang Z, Wu Y, Liu D, Zhang W, Wu Q, Chen Y (2022). Mo_2_N quantum dots decorated N-doped graphene nanosheets as dual-functional interlayer for dendrite-free and shuttle-free lithium–sulfur batteries. Adv. Func. Mater..

[171] Jing W, Zu J, Zou K, Dai X, Song Y, Han J, Sun J, Tan Q, Chen Y, Liu Y (2022). Sandwich-like strontium fluoride graphene-modified separator inhibits polysulfide shuttling and lithium dendrite growth in lithium–sulfur batteries. J. Mater. Chem. A.

[172] Chen L, Sun Y, Wei X, Song L, Tao G, Cao X, Wang D, Zhou G, Song Y (2023). Dual-functional V_2_C MXene assembly in facilitating sulfur evolution kinetics and Li-ion sieving toward practical lithium–sulfur batteries. Adv. Mater..

[173] Hou Y, Huang Z, Chen Z, Li X, Chen A, Li P, Wang Y, Zhi C (2022). Bifunctional separators design for safe lithium-ion batteries: suppressed lithium dendrites and fire retardance. Nano Energy.

[174] Wang J, Yi S, Liu J, Sun S, Liu Y, Yang D, Xi K, Gao G, Abdelkader A, Yan W, Ding S, Kumar RV (2020). Suppressing the shuttle effect and dendrite growth in lithium–sulfur batteries. ACS Nano.

[175] Yu X, Liao T, Tang J, Zhang K, Tang S, Gao R-S, Lin S, Qin L-C (2021). Edge engineering in 2D molybdenum disulfide: simultaneous regulation of lithium and polysulfides for stable lithium–sulfur batteries. Adv. Energy Sustain. Res..

[176] Shi H, Qin J, Huang K, Lu P, Zhang C, Dong Y, Ye M, Liu Z, Wu ZS (2020). A two-dimensional mesoporous polypyrrole-graphene oxide heterostructure as a dual-functional ion redistributor for dendrite-free lithium metal anodes. Angew. Chem..

[177] Mao Y-Q, Dong G-H, Zhu W-B, Li Y-Q, Huang P, Fu S-Y (2023). Novel sandwich structured glass fiber cloth/poly(ethylene oxide)-MXene composite electrolyte. Nano Mater. Sci..

[178] Tang Y, Xiong Y, Wu L, Xiong X, Me T, Wang X (2023). A solid-state lithium battery with PVDF–HFP-modified fireproof ionogel polymer electrolyte. ACS Appl. Energy Mater..

[179] Liu P, Wang X, Jia X, Zhou J (2022). Carbon-confined two-dimensional sodiophilic sites boosted dendrite-free sodium metal anodes. ACS Appl. Mater. Interfaces.

[180] Matios E, Wang H, Wang C, Hu X, Lu X, Luo J, Li W (2019). Graphene regulated ceramic electrolyte for solid-state sodium metal battery with superior electrochemical stability. ACS Appl. Mater. Interfaces.

[181] Zhang L, Zhu X, Wang G, Xu G, Wu M, Liu HK, Dou SX, Wu C (2021). Bi nanoparticles embedded in 2D carbon nanosheets as an interfacial layer for advanced sodium metal anodes. Small.

[182] Li Z, Zhang Y, Guan H, Meng S, Lu Y, Wang J, Huang G, Li X, Cui J, Li Q, Zhang Q, Qu B (2023). Rationally integrating 2D confinement and high sodiophilicity toward SnO_2_/Ti_3_C_2_T_x_ composites for high-performance sodium-metal anodes. Small.

[183] Fang Y, Zhang Y, Zhu K, Lian R, Gao Y, Yin J, Ye K, Cheng K, Yan J, Wang G, Wei Y, Cao D (2019). Lithiophilic three-dimensional porous Ti_3_C_2_T_x_–rGO membrane as a stable scaffold for safe alkali metal (Li or Na) anodes. ACS Nano.

[184] Wang G, Yu F, Zhang Y, Zhang Y, Zhu M, Xu G, Wu M, Liu H-K, Dou S-X, Wu C (2021). 2D Sn/C freestanding frameworks as a robust nucleation layer for highly stable sodium metal anodes with a high utilization. Nano Energy.

[185] He X, Jin S, Miao L, Cai Y, Hou Y, Li H, Zhang K, Yan Z, Chen J (2020). A 3D hydroxylated MXene/carbon nanotubes composite as a scaffold for dendrite-free sodium-metal electrodes. Angew. Chem..

[186] Go W, Kim MH, Park J, Lim CH, Joo SH, Kim Y, Lee HW (2019). Nanocrevasse-rich carbon fibers for stable lithium and sodium metal anodes. Nano Lett..

