# Correction: Two-dimensional materials for high density, safe and robust metal anodes batteries

**DOI:** 10.1186/s40580-023-00394-2

**Published:** 2023-09-22

**Authors:** Hoilun Wong, Yuyin Li, Jun Wang, Tsz Wing Tang, Yuting Cai, Mengyang Xu, Hongliang Li, Tae-Hyung Kim, Zhengtang Luo

**Affiliations:** 1 Department of Chemical and Biological Engineering and William Mong Institute of Nano Science and Technology, Hong Kong University of Science and Technology, Clear Water Bay, Kowloon, Hong Kong; 2 Hefei National Research Center for Physical Sciences at the Microscale, University of Science and Technology of China, Hefei, Anhui People’s Republic of China; 3 School of Integrative Engineering, Chung-Ang University, Seoul, 06974 Republic of Korea

**Correction: Nano Convergence (2023) 10:37** 10.1186/s40580-023-00384-4

Following publication of the original article [1], the authors noticed that a footnote denoting equal contribution was omitted and the affiliations for the corresponding author are given incorrectly. The following footnote should have been included for authors “Hoilun Wong and Yuyin Li contributed equally to this work.” The corresponding author “Zhengtang Luo” is affiliated only in “Department of Chemical and Biological Engineering and William Mong Institute of Nano Science and Technology, Hong Kong University of Science and Technology, Clear Water Bay, Kowloon, Hong Kong”. These correction do not affect any results or conclusions of the publication.

The original article has been corrected.
